# A Bidirectional Mendelian Randomization Study to evaluate the causal role of reduced blood vitamin D levels with type 2 diabetes risk in South Asians and Europeans

**DOI:** 10.1186/s12937-021-00725-1

**Published:** 2021-07-27

**Authors:** Cynthia A. Bejar, Shiwali Goyal, Shoaib Afzal, Massimo Mangino, Ang Zhou, Peter J. van der Most, Yanchun Bao, Vipin Gupta, Melissa C. Smart, Gagandeep K. Walia, Niek Verweij, Christine Power, Dorairaj Prabhakaran, Jai Rup Singh, Narinder K. Mehra, Gurpreet S. Wander, Sarju Ralhan, Sanjay Kinra, Meena Kumari, Martin H. de Borst, Elina Hyppönen, Tim D. Spector, Børge G. Nordestgaard, Piers R. Blackett, Dharambir K. Sanghera

**Affiliations:** 1 Department of Pediatrics, Section of Genetics, College of Medicine, University of Oklahoma Health Sciences Center, 940 Stanton L. Young Blvd., Rm 317 BMSB, OK 73104 OK City, USA; 2 Department of Clinical Biochemistry, Herlev and Gentofte Hospital, Copenhagen University Hospital, Herlev, Denmark; 3 The Copenhagen General Population Study, Herlev and Gentofte Hospital, Copenhagen University Hospital, Herlev, Denmark; 4 Faculty of Health and Medical Sciences, University of Copenhagen, Copenhagen, Denmark; 5 Department of Twin Research and Genetic Epidemiology, Kings College London, London, SE1 7EH UK; 6 NIHR Biomedical Research Centre at Guy’s and St Thomas’ Foundation Trust, SE1 9RT London, UK; 7 Australian Center for Precision Health, University of South Australia Cancer Research Institute, Adelaide, Australia; 8 Department of Epidemiology, University of Groningen, University Medical Center Groningen, Groningen, NL The Netherlands; 9 Department of Mathematical Sciences, University of Essex, Colchester, UK; 10 Department of Anthropology, University of Delhi, New Delhi, India; 11 Public Health Foundation of India, Gurgaon, India; 12 Department of Internal Medicine, Division of Nephrology, University of Groningen, University Medical Center Groningen, Groningen, The Netherlands; 13 Population, Policy and Practice, Institute of Child Health, University College London, London, WC1N 1EH UK; 14 Department of Human Genetics, Central University of Punjab, Bathinda, Punjab India; 15 Department of Transplant Immunology and Immunogenetics, All India Institute of Medical Sciences and Research, New Delhi, India; 16 Department of Cardiology, Hero DMC Heart Institute, Ludhiana, India; 17 Department of Non-Communicable Disease Epidemiology, London School of Hygiene and Tropical Medicine, London, UK; 18 Australian Centre for Precision Health, South Australian Health and Medical Research Institute, Adelaide, Australia; 19 Department of Pediatrics, Section of Pediatric Endocrinology, College of Medicine, University of Oklahoma Health Sciences Center, Oklahoma City, OK USA; 20 Harold Hamm Diabetes Center, University of Oklahoma Health Sciences Center, Oklahoma City, OK USA; 21 Department of Pharmaceutical Sciences, University of Oklahoma Health Sciences Center, Oklahoma City, OK USA; 22 Department of Physiology, University of Oklahoma Health Sciences Center, Oklahoma City, OK USA; 23 Oklahoma Center for Neuroscience, University of Oklahoma Health Sciences Center, Oklahoma City, OK USA

## Abstract

**Context:**

Multiple observational studies have reported an inverse relationship between 25-hydroxyvitamin D concentrations (25(OH)D) and type 2 diabetes (T2D). However, the results of short- and long-term interventional trials concerning the relationship between 25(OH)D and T2D risk have been inconsistent.

**Objectives and methods:**

To evaluate the causal role of reduced blood 25(OH)D in T2D, here we have performed a bidirectional Mendelian randomization study using 59,890 individuals (5,862 T2D cases and 54,028 controls) from European and Asian Indian ancestries. We used six known SNPs, including three T2D SNPs and three vitamin D pathway SNPs, as a genetic instrument to evaluate the causality and direction of the association between T2D and circulating 25(OH)D concentration.

**Results:**

Results of the combined meta-analysis of eight participating studies showed that a composite score of three T2D SNPs would significantly increase T2D risk by an odds ratio (OR) of 1.24, p = 1.82 × 10^–32^; Z score 11.86, which, however, had no significant association with 25(OH)D status (Beta -0.02nmol/L ± SE 0.01nmol/L; p = 0.83; Z score -0.21). Likewise, the genetically instrumented composite score of 25(OH)D lowering alleles significantly decreased 25(OH)D concentrations (-2.1nmol/L ± SE 0.1nmol/L, p = 7.92 × 10^–78^; Z score -18.68) but was not associated with increased risk for T2D (OR 1.00, p = 0.12; Z score 1.54). However, using 25(OH)D synthesis SNP (DHCR7; rs12785878) as an individual genetic instrument, a per allele reduction of 25(OH)D concentration (-4.2nmol/L ± SE 0.3nmol/L) was predicted to increase T2D risk by 5%, p = 0.004; Z score 2.84. This effect, however, was not seen in other 25(OH)D SNPs (GC rs2282679, CYP2R1 rs12794714) when used as an individual instrument.

**Conclusion:**

Our new data on this bidirectional Mendelian randomization study suggests that genetically instrumented T2D risk does not cause changes in 25(OH)D levels. However, genetically regulated 25(OH)D deficiency due to vitamin D synthesis gene (DHCR7) may influence the risk of T2D.

**Supplementary Information:**

The online version contains supplementary material available at 10.1186/s12937-021-00725-1.

## Introduction

Type 2 diabetes mellitus (T2D) has become a global health epidemic of twenty-first century. The International Diabetes Federation (IDF) data showed that the number of people affected with T2D would rise from 382 million in 2011 to 592 million by 2035 [1]. T2D is a complicated disease impacted by the complex interplay of genetic, epigenetic, and environmental factors [2–4]. In addition, several lifestyle factors such as sedentary lifestyle, westernized diet, smoking, and genetic predisposition enhance T2D risk in some ethnic groups [5, 6].

Vitamin D deficiency increases in conjunction with T2D, Type 1 diabetes mellitus (T1D), obesity, and cardiovascular disease [7]. The inverse relationship between circulating 25(OH)D concentrations and T2D is confirmed in several observational and prospective studies from the US, Australia, Europe, and Asia [8–11]. However, a causal link has not been established between low blood 25(OH)D levels and T2D risk. Recently, a large clinical trial of vitamin D3 supplementation on 2423 participants for 2.5 years concluded that a dose of 4000 IU of 25(OH)D3 did not protect people from developing T2D [12]. Findings from observational studies may be subject to residual confounding because it is difficult to measure individual variation specifically related to sun exposure and cultural variations in such studies. It may also be challenging to rule out the reverse causality accounting for any association. On the other hand, human genetic information is used by the methodology of Mendelian randomization, which takes account of genetic instruments to provide an unconfounded estimate of the association. The Mendelian randomization strategy is based on the principle that the individual genotypes are randomly assigned and can be used as a genetic instrument with the assumption that their involvement affects the outcome only by modifying the biomarkers (i.e., circulating 25(OH)D) and can also be used to test the direction of causation [13, 14].

The blood level of 25(OH)D is the best and commonly used method to determine vitamin D status. According to the US Endocrinology Society, vitamin D deficiency is defined as a 25(OH)D level of ≤ 20 ng/mL; insufficiency as 21 to 29 ng/mL; and sufficiency as > 30 ng/mL or more [15]. Low 25(OH)D status (deficiency or insufficiency) is highly prevalent among elderly, postmenopausal women, and certain ethnic population groups and is influenced by both genetic and environmental factors [16–19]. Mainly, populations due to specific ethnic backgrounds (South Asian, East Asian, and African) show a high prevalence of blood 25(OH)D deficiency [10, 20, 21]. Genetically regulated 25(OH)D concentration in a longitudinal follow-up study of 95,766 Europeans of Danish ancestry showed that reduced 25(OH)D levels were associated with all-cause mortality, cancer, and mortality from other causes [11].

Common genetic variants in ~ six loci have been identified to affect blood 25(OH)D concentrations in genome-wide association studies (GWAS) performed in European whites [22], Hispanics [23], and Asian Indians [24]. Also, multiple GWAS on T2D have identified the association of common variants in over 100 genes in ethnically diverse cohorts [4]. However, whether genetically influenced reduced 25(OH)D concentrations can be causal or contributes to enhancing T2D risk, or conversely, whether gene variants that are now widely accepted to be robustly associated with increased risk for T2D could be involved in altering blood 25(OH)D levels is unclear and inconsistent.

Here, we have used genetic instrumental variable methods to obtain estimates of the causal association between circulating 25(OH)D concentrations and T2D, including the direction of causality, by performing two-directional reciprocal Mendelian randomization study [25]. The selection of index SNPs from six genetic loci as an instrumental variable was based on the bonafide gene-regions identified in GWAS and metanalysis studies for affecting T2D and blood 25(OH)D concentrations and their availability in all participant studies [22, 24, 26–28]. These included two clusters of 3 index SNPs, each associated with 25(OH)D concentrations (GC rs2282679, DHCR7 rs12785878, CYP2R1 rs12794714), and T2D (IGF2BP2 rs1470579, TCF7L2 rs7903146, and KCNQ1 rs2237896), respectively. Even though multiple GWAS studies have pointed out the role of variants in genes involved in the synthesis, transport, and metabolism for influencing circulating 25(OH)D concentrations, the results of earlier published Mendelian randomization studies to determine the causal link between vitamin D insufficiency and T2D risk have been inconsistent and controversial. With few exceptions, these studies neither clearly support nor exclude the causal association of 25(OH)D levels with T2D [11, 29–31]. Moreover, these studies have examined mainly individuals from European populations, except one recent study included East Asians with Europeans [31]. Thus, in this study, we, for the first time, have included data from South Asian Indians with Europeans. People from the Indian sub-continent have 3 to sixfold higher T2D prevalence than Europeans and are a highly understudied population for vitamin D genetic studies. There is a paucity of data showing the relationship between vitamin D status and T2D in South Asian Indians [32].

## Research design and methods

### Ethical statement

All participating studies were reviewed and approved by the respective Universities or Institutional Review Boards, including the primary institute of the University of Oklahoma Health Sciences Center’s Institutional Review Board, as well as the Human Subject Protection Committees at the participating hospitals and institutes in India. All participants provided written informed consent for investigations.

### Study design and phenotypic measurements

Our study with data from up to 59,890 participants from eight cohorts included: the Asian Indian Diabetic Heart Study/Sikh Diabetes Study (AIDHS/SDS); the Indian Migration Study (IMS); Twins UK; The British 1958 birth cohort (1958 BC); the Copenhagen City Heart Study (CCHS), the Copenhagen City General Population Study (CGPS), and the Copenhagen Ischemic Heart Disease Study (CIHDS); The UK Household Longitudinal Study (UKHLS); and The Prevention of Renal and Vascular End-Stage Disease (PREVEND) study. Details of study design and experimental work flow is presented in the Flow Chart (online Supplementary Section). Phenotypic measurements including T2D, 25(OH)D and other recruitment details are presented separately for each cohort (online Supplementary Section). Measures of blood 25(OH)D levels were not available on UKHLS (European) and MS India (Asian Indian) cohorts. UV index values were calculated for the city nearest the blood draw location and the month of recruitment using data from the National Weather Service Climate Prediction Center (http://www.cpc.ncep.noaa.gov) and by averaging of the previous three months UV index as described previously [33]. The clinical characteristics of each study cohort stratified by T2D are summarized in Table 1.

**Table 1:** Clinical and demographic traits of subjects in the participating cohorts stratified by type 2 diabetes

**Study Cohorts**	**Location**	**Type 2 Diabetes**	**N**	**Female**	**Age**	**BMI**	**25(OH)D**
				%	(yrs)	(kg/m2)	(nmol/L)
**South Asian**							
AIDHS/SDS	Punjab, India	Cases	1586	44	54.0±11.1	27.4±4.9	31.2±37.6
STAGE I		Controls	1123	46	51.0±14.3	26.5±4.9	46.3±45.5
		Combined	2709	45	52.8±12.6	27.0±4.9	37.0±41.5
AIDHS/SDS	Punjab, India	Cases	1164	44	55.9±11.9	27.1±4.8	32.8±21.4
STAGE II		Controls	1033	44	46.2±14.2	25.8±4.5	34.9±23.8
		Combined	2197	44	51.4±13.9	26.5±4.7	33.8±22.5
	Andra Pradesh India	Cases	385	35	44.2±0.5	23.8±0.2	N/A
IMS^†^		Controls	485	37	47.5±0.3	26.0±0.2	N/A
		Combined	870	36	46.0±8.4	25.0±4.2	N/A
**Caucasian**							
	London	Cases	124	100	61.1±9.4	28.1±5.9	76.2±44.9
TWINS UK	UK	Controls	5215	91	52.1±14.5	26.1±4.9	76.4±40.5
		Combined	5339	91	52.3±14.4	26.1±5.0	76.4±40.6
	England/Scotland/Wales	Cases	179	41	45^*^	33.9±6.7	47.4±23.4
1958 Birth Cohort	UK	Controls	5002	50	45^*^	27.2±4.7	57.9±25.5
		Combined	5181	50	45^*^	27.4±4.9	57.6±25.5
	Copenhagen, Denmark	Cases	1562	44	65.8±10.1	29.5±5.1	47.3±25.0
CCHS/CGPS/ CIHDS		Controls	29478	56	59.2±12.9	25.9±4.2	54.1±26.0
		Combined	31040	55	59.5±12.8	26.1±4.3	53.7±26.0
	Essex, UK	Cases	496	45	64.9±12.3	31.8±6.1	N/A
UKHLS		Controls	8409	57	52.6±16.1	27.9±5.1	N/A
		Combined	8905	56	53.3±16.2	28.1±5.3	N/A
	Groningen,	Cases	366	62	56.5±10.1	29.4±4.7	55.1±21.0
PREVEND	Netherland	Controls	3283	50	49.5±12.5	25.8±4.0	59.0±23.7
		Combined	3649	52	50.2±12.5	26.1±4.3	58.6±23.4

^†^Total 435 sib pairs (870 individuals); 54.8% T2D (*N* = 870); Out of 435 pairs, 385 pairs are discordant for T2D, and 50 pairs are concordant for T2D

^*^Please note all participants are at the same age. Information for BMI, blood 25(OH)D were measured from 45 yr. survey, whereas T2D cases/controls were ascertained from 45, 46 and 50 yr. surveys combined

Abbreviations: N/A: Not available, AIDHS/SDS: Asian Indian Diabetic Heart Study/ Sikh Diabetes Study, IMS: Indian Migration Study, CCHS/CGPS/CIHDS: Copenhagen City Heart Study/ Copenhagen General Population Study/ Copenhagen Ischemic Heart Disease Study, UKHLS: UK Household Longitudinal Study, PREVEND: Prevention of Renal and Vascular End-Stage Disease study

### SNP Genotyping and Quality Control (QC) and statistical analysis

The selection of SNPs as genetic instruments was based on their strong global association with respective exposure phenotypes of T2D and 25(OH)D reported in multiethnic GWAS and meta-analysis studies [22, 26–28]. Additional criteria for selecting instrumental variables were based on their availability in most of the participating studies. We have provided genotyping, sample, and SNP QC details separately by each cohort in the online Supplementary Section. Genome-wide genotype data for each cohort were checked for population structure using principal components, and outliers were removed before the analysis. To ensure that the Mendelian randomization assumptions were not violated, all SNPs selected as genetic instruments were strongly associated with the outcome (i.e. gene variants associated with T2D for increasing T2D outcome; and gene variants associated with vitamin D for affecting plasma 25(OH)D outcome). None of the SNPs used as Mendelian instruments for T2D and 25(OH)D were in linkage disequilibrium (*r*^2^ = 0.001). None of the instrumental variables were associated with the outcome via exposure to other factors (BMI, age, and gender).

For 25(OH)D analysis, linear regression and additive genetic model were used with the natural log-transformed 25(OH)D level adjusted with age, gender, BMI, and study-specific covariates (e.g., T2D, UV index). Similarly, the three GWAS SNPs known to affect T2D risk were analyzed for their association with T2D using logistic regression and an additive genetic model. The T2D binary trait was the dependent variable with covariates age, gender, BMI. Each cohort analysis was limited to a single ancestral group (e.g. European ancestry only, South Asian only). If selected SNPs were unavailable, the best proxy SNP (SNP on the local haplotype having strong linkage disequilibrium (LD) with the reference SNP) was used using SNAP [34].

Upon completing the association analysis, the final list of SNPs was determined for gene score construction for each trait. The SNP, risk allele, number of risk alleles, and effect size (of each allele regarding the effect allele for log-transformed 25(OH)D levels) were required to construct a weighted gene score using the PLINK program [35]. Samples with missing 25(OH)D levels were excluded from the analysis (see Supplementary Section). A weighted gene score construction for T2D required the SNP, risk allele, number of risk alleles, and odds ratio (of each allele concerning the effect allele for T2D) for gene construction through PLINK as described previously [22]. Samples missing T2D status data were excluded, and SNPs that were directly genotyped are included to construct both gene scores. The additional parameter “–score-no-mean-imputation” was used in PLINK to construct both gene scores. The “–score-no-mean-imputation” was used so that the missing allele was not accounted as the most common allele based on the sample allele frequencies while constructing gene scores. The constructed gene scores were each normalized to a quantitative variable at an appropriate scale.

An association analysis was performed on the constructed gene scores for each cohort. A linear regression and an additive genetic model were used with blood 25(OH)D levels as the dependent variable adjusted by gender, age, BMI, and T2D. Similarly, a logistic regression and an additive genetic model were used with T2D as the dependent variable adjusted by the gender, age, BMI. To combine the association analysis results of each trait by SNP for each cohort, a fixed effect inverse variance meta-analysis implemented in METAL [36] was performed. This required the minor allele frequency, effect size, standard error, and p-value for each SNP across the different ethnic cohorts. The combined results were stratified by their respective trait association; the three SNPs associated with 25(OH)D levels were assessed for their association with T2D and 25(OH)D. Similarly, the three significant SNPs for T2D were evaluated for their association with T2D and blood 25(OH)D. Each cohort provided their statistical data summaries for the SNPs, and meta-analysis was performed on these SNPs by trait using METAL and Forest Plot Viewer (http://ntp.niehs.nih.gov/ntp/ohat/forestplot/), and PRISM (https://www.graphpad.com/scientific-software/prism/) was used to generate the forest plots. We did not observe heterogeneity of association of the genetic instruments of T2D and 25(OH)D with their respective phenotypes between studies; thus, fixed-effect models were used in metanalysis to drive odds ratios and confidence intervals. Finally, to assess the robustness of our conclusions, we applied conservative Bonferroni’s correction and used a corrected significant threshold of 0.0063 (0.05/ 8 test variables including six instrument SNPs, T2D, and 25(OH)D concentration)

## Results

Demographics and clinical characteristics of all study cohorts are summarized in Table 1. As expected, there were significant differences in the distribution of BMI and 25(OH)D among cases and controls in most participating cohorts. The overall distribution of blood 25(OH)D was consistent with previously published reports on these studies, and mean 25(OH)D levels were much lower across South Asian, compared to European cohorts. Data on 25(OH)D concentration was available for only a few individuals in the South Asian cohorts, except for the Punjabi Sikh studies (AIDHS/SDS).

### Association of T2D susceptibility genes with T2D and blood 25(OH)D concentrations

The association of genetic variants with an increased risk for T2D was analyzed using three known and well-established T2D associated variants from previous studies. Meta-analysis of association of IGF2BP2 (rs1470579) with T2D (adjusted for age, gender, and BMI) showed an OR (95% CI) of 1.12 (1.08, 1.16), *P*= 7.2x10^-7^, and Z score = 4.96 (Supplementary Table 1). Similarly, the adjusted per- allele effect for TCF7L2 (rs7903146) was associated with an increased risk for T2D (OR 1.25 (95% CI (1.23, 1.28), *P*= 1.3 x10^-32^, and Z score = 11.31 (Supplementary Table 1). Similarly, the adjusted per-allele effect of KCNQ1 (rs2237896) was 1.49 (1.43, 1.55), *P*= 5.07 x10^-7^, and Z score = 5.02 (Supplementary Table 1). We then analyzed the same T2D variants for their association with blood 25(OH)D concentrations in the same population cohorts. None of the variants associated with increased or decreased risk for T2D showed any association for affecting blood 25(OH) D concentrations. The per-allele association of IGF2BP2 (rs1470579) for affecting blood 25(OH)D concentration was Beta ± SE (0.0058, 0.005) *P*= 0.20, Z score = 1.29 (Supplementary Table 2). The per allele effect for TCF7L2 (rs7903146) for affecting blood 25(OH)D concentration was marginally significant Beta ± SE (-0.011, 0.005), *P*= 0.02, Z score = -2.30, and no association was observed in KCNQ1 (rs2237896) with 25(OH)D concentration Beta ± SE (-0.005, 0.01]), *P*= 0.26, Z score = -1.18 after adjusting for age, gender, and T2D (Supplementary Table 2).

### Genes involved in 25(OH)D synthesis, metabolism, and transport and their effects on 25(OH)D concentrations and T2D

Interestingly, not only was there a significant association, the direction of the minor (effect) allele of GC (rs2282679) and CYP2R1 (rs12794714) and DHCR7 (rs12785878) with blood 25(OH)D were largely consistent across chorts (Supplementary Table 3). The minor (effect) allele frequency was significantly higher in South Asian (0.73 Sikhs) populations compared to Caucasian cohortso (which ranged between 0.22-0.32) (Supplementary Table 3). In the combined meta-analysis of 30,058 samples, the per allele effect of the GC variant (rs2282679) for its association with 25(OH)D levels was (Beta -0.091 ± SE 0.005); *P*= 2.87x10^-61^; and Z score = -16.52 nmol/L after adjusting for age, gender and BMI. For CYP2R1, age, gender, and BMI adjusted association was (Beta -0.039 ± SE 0.0032); *P*=7.56x10^-34^; and Z score -11.66 nmol/L, while the association of the DHCR7 variant (rs12785878) resulted in Beta -0.042 ± SE 0.0032, *P*= 9.0x10^-32^; and Z score -11.73 nmol/L for a total of 49,433 samples (Supplementary Table 3).

Next, we tested the association of the same variants with the risk of T2D. As summarized in Supplementary Table 4, neither variants for GC and CYP2R1 were associated with the risk of increasing T2D in any of the individual cohorts in the combined meta-analysis. The per-allele effect for increasing T2D risk yielded an OR (95% CI’s) of 1.03 (0.97,1.09), *P*=0.207, and Z score = 0.16 for the GC variant (rs2282679), and 1.01 (0.97,1.05), *P*=0.453, Z score = 1.09 for the CYP2R1 (rs12794714). However, the per-allele effect of the DHCR7 (rs12785878) variant showed a significantly increased risk for T2D OR 1.05 95% CI (1.0, 1.11), *P*=0.004, and Z score =2.84 in the meta-analysis (Supplementary Table 4). We also adjusted the effects of UV index on modulating association of 25(OH)D SNPs on 25(OH)D concentrations (Supplementary Tables 5), and also for their association with T2D (Supplementary Tables 6). The overall outcome of association remained unchanged as the data on the UV index was only available in AIDHS/SDS.

### Gene score association analysis

The gene score construction for SNP alleles associated with an increased risk for T2D, IGF2BP2 (rs1470579), TCF7L2 (rs7903146), and KCNQ1 (rs2237896) showed a significant association with increased risk for T2D in all studies OR (95% CI) 1.24 (1.22, 1.26), P= 1.82x10^-32^, Z score 11.86 in 51,816 subjects (Table 2; Supplementary Figure 1A). However, the same allelic score showed no association for affecting blood 25(OH)D concentration (Beta ± SE [-0.0002 ± 0.0001], *P*= 0.829, Z score = -0.212). These results appear to suggest that diabetes genetic loci increase T2D risk independently without modulating 25(OH)D concentrations (Table 3 and Supplementary Figure 1B).

**Table 2:** Association analysis of composite gene score of T2D SNPs (IGF2BP2, TCF7L2, and KCNQ1) used as a genetic instrument for their joint effect on T2D

Study	N	OR	LCI	UCI	P Val	Z score	Direction
AIDHS/SDS	2675	1.42	1.33	1.52	6.09E-13		
SIKH REPLICATION	2197	1.69	1.36	2.02	2.00E-03		
IMS	848	1.27	1.04	1.51	4.62E-02		
**SOUTH ASIAN META**	**5720**	**1.42**	**1.34**	**1.50**	**1.33E-12**	**7.09**	** + + + **
Twins UK	5335	1.18	1.00	1.35	6.84E-02		
1958 BC	5181	1.30	1.13	1.46	1.81E-03		
CCHS/CGPS/CIHDS	23,029	1.24	1.22	1.26	5.70E-15		
UKHLS	8902	1.18	1.09	1.26	1.53E-04		
PREVEND	3649	1.27	1.17	1.37	2.18E-06		
**Meta-Analysis**	**51,816**	**1.24**	**1.22**	**1.26**	**1.82E-32**	**11.86**	** + + + + + + + + **

**Table 3:** Association analysis of composite gene score of T2D SNPs (IGF2BP2, TCF7L2 and KCNQ1) used as a genetic Instrument for their joint effects on 25(OH)D concentrations

Study	N	Beta	SE	P Val	Z Score	Direction
AIDHS/SDS	2675	-0.006	0.014	6.98E-01		
SIKH REPLICATION	2197	-0.001	0.009	8.96E-01		
**SOUTH ASIAN META**	**4872**	**-0.003**	**0.008**	**7.45E-01**	**-0.367**	**– –**
Twins UK	5335	0.0004	0.001	7.07E-01		
1958 BC	5181	0.1322	0.056	1.90E-02		
CCHS/CGPS/CIHDS	11,665	-0.0080	0.007	2.35E-01		
PREVEND	3649	-0.0154	0.031	6.20E-01		
**Meta-Analysis**	**30,702**	**-0.0002**	**0.0001**	**8.29E-01**	**-0.212**	**– – + + – –**

Next, we combined the allelic effects of genes affecting blood 25(OH)D concentrations and gene variants associated with T2D risk by constructing a gene score. Table 4 shows a significant association between the gene score of 25(OH)D lowering alleles in GC (rs2282679), CYP2R1 (rs12794714), and DHCR7 (rs12785878) in a total of 41,136 individuals which yielded Beta ± SE (-0.021 ± 0.001), i= 7.92x10^-78^, and Z score = -18.68 (Supplementary Figure 2A). However, the same gene score of the 25(OH)D lowering allele was not associated with the increased risk for T2D OR, 1.002 (1.001-1.005), *p*= 0.12, and Z score = 1.54 (Table 5 and Supplementary Figure 2B).

**Table 4:** Association analysis of composite gene score of vitamin D SNPs (GC, CYP2R1 and DHCR7) used as a genetic in strument for their effect on 25(OH)D concentrations

Study	N	Beta	SE	P Val	Z score	Direction
AIDHS/SDS	2388	-0.042	0.012	2.70E-04		
SIKH REPLICATION	1846	-0.025	0.009	4.00E-03		
**SOUTH ASIAN META**	**4234**	**-0.031**	**0.007**	**4.01E-06**	**-4.61**	**– –**
Twins UK	3679	-0.129	0.020	5.20E-11		
1958 BC	4993	-0.035	0.005	4.20E-14		
CCHS/CGPS/CIHDS	24,581	-0.020	0.001	3.00E-39		
PREVEND	3649	-0.021	0.002	3.70E-33		
**Meta-Analysis**	**41,136**	**-0.021**	**0.001**	**7.92E-78**	**-18.68**	**– – – – – –**

**Table 5:** Association analysis of composite gene score of vitamin D SNPs (GC, CYP2R1 and DHCR7) used as a genetic Instrument for their association with T2D

VitD_T2D	N	OR	LCI	UCI	P_Val	Z score	Direction
AIDHS/SDS	2388	1.004	1.00	1.01	2.30E-02		
SIKH REPLICATION	1846	1.002	1.00	1.01	4.11E-01		
**SOUTH ASIAN META**	**4234**	**1.003**	**1.00**	**1.01**	**2.90E-02**	**2.19**	** + + **
Twins UK	3679	0.89	0.56	1.42	6.26E-01		
1958 BC	4993	1.00	0.99	1.05	7.92E-01		
CCHS/CGPS/CIHDS	28,372	1.00	0.99	1.004	6.19E-01		
PREVEND	3649	1.00	0.98	1.03	6.79E-01		
**Meta-Analysis**	**44,927**	**1.002**	**1.001**	**1.005**	**1.20E-01**	**1.54**	** + + – + + + **

## Discussion

Here we performed a Mendelian randomization study to investigate whether genetically reduced vitamin D levels could be causally related to increasing T2D risk using data from up to 59,890 participants (5,862 cases and 54,028 controls). Essentially, our findings suggest that even though the candidate genetic variants (individually) or via composite gene score are associated with increased susceptibility to T2D, these are not involved in affecting blood 25(OH)D concentration. This means that the genetically instrumented T2D risk could be independent of the pathway (s) linked with vitamin D synthesis, transport, or early stages of metabolism. Additionally, our data showed that the genetically instrumented reduction of 25(OH)D levels does not increase T2D susceptibility in a composite gene score. More specifically, gene variants in the vitamin D transporter (GC) and metabolism (hydroxylation) (CYP2R1) regulate 25(OH)D concentrations independent of influencing T2D risk. On the other hand, in the (DHCR7; the gene involved in 25(OH)D synthesis), the genetically instrumented decrease of -4.2 nmol/L ± 0.3 nmol/L SE of 25(OH)D concentration (Supplementary Table 3) was predicted to increase T2D risk by 5% [95% CI 0%,11%], *p* = 0.004, with a Z score of 2.84 (Supplementary Table 4). These results agree with an earlier published study on 96,423 Danes (one of the participating cohorts from Copenhagen) in which the DHCR7 allelic effect was associated with a modestly increased risk of T2D (OR 1.51 [95% CI 0.98, 2.33]; *p* trend = 0.04), whereas no association was observed for CYP2R1 (OR 1.02 [95% CI 0.75, 1.37]; *p* = 0.86) in that study [11].

During exposure to sunlight, a precursor molecule, 7-dehydrocholesterol (DHC), is converted to cholesterol by the action of ultraviolet light (UV) on the skin through a thermal isomerization. Hydroxylation of pre-vitamin D3 occurs in the liver from the actions of CYPs (CYP2R1). The CYP27A1 drives the conversion of 25(OH)D to 1,25(OH)D in the kidney (Supplementary Fig. 3). While our present study was in progress, a study using the Mendelian randomization approach showed that a combined gene score of 25(OH)D genes involved in synthesis (DHCR7 rs12785878) and metabolism (CYP2R1 rs10741657) was associated with an increased risk for T2D in European and Chinese adults [31]. However, our results could not confirm the role of CYP2R1 variants in the risk for T2D. This discrepancy could be due to the use of a different CYP2R1 SNP (rs12794714) in the present study or due to the overlapping role of multiple cytochrome P450 (CYP) enzymes on the hydrolysis of 25(OH)D in hepatocytes [37].

As multiple CYPs in addition to CYP2R1 and CYP27A1 could evidently be involved in vitamin D hydroxylation in the liver [38]. Therefore, the role of other CYPs resulting in compensation for 25 hydroxylase activity cannot be ruled out [37]. Thus, in light of these findings, our negative findings on genetically reduced vitamin D by CYP2R1 are not surprising.

Presumably, the differences in linkage disequilibrium patterns among different populations, population-specific risk factors, and pleiotropy may have masked the potential cumulative effects of gene scores. It appears that perhaps due to similar limitations, the study of Lu et al*.* [31] also could not capture the impact of genetically regulated 25(OH)D concentration on T2D using a genetically instrumented gene score of four SNPs GC (rs2282679), CYP2R1 (rs10741657), Cyp27A1 (rs6013897) and DHCR7 (rs12785878) with T2D (*p* = 0.07) in meta-analysis despite using a sample size of up to 476,099 of Chinese and European adults.

We undertook an additional meta-analysis combining the results of our analyses with those of Lu et al*.* [31]. These findings for the DHCR7 SNP showed that the genetically instrumented reduction in 25(OH)D of Beta ± SE (-4.3 nmol/L ± 0.3 nmol/L), *p* = 1.26 × 10^–98^, Z score -21.08 (Supplementary Table 7B) would significantly increase T2D risk from 5% (current study) to 7%; *P* = 5 × 10^–4^ Z score = 3.51 in a total of 493,057 individuals (Supplementary Table 8B). However, we did not observe this effect in the meta-analysis of the GC variant with the results of Lu et al. [31] (Supplementary Tables 7A and 8A). These data further confirm the possible causal effects associated with synthesis (DHCR) SNPs for increasing the risk of T2D.

The strengths of our study include well-characterized multiethnic cohorts to detect allelic association of vitamin D genes with T2D risk. We reduced the confounding effects of age, gender, and obesity across all studies. Principal components were used in each individual cohort to control for the potential of confounding by population stratification. Sample size–weighted Z score method was used to reduce inter-study variation in the diverse ancestries and differences in 25(OH)D assays between cohorts as described earlier [22]. Limitations of this study include insufficient data on 25(OH)D measures and T2D phenotypes in some cohorts; over-representation of European cohorts; lack of data on 25(OH)D measures in most South Asian studies that were available with GWAS; and data access was limited to summary statistics for each study cohorts. It is possible that the differences in serum 25(OH)D levels and T2D prevalence between South Asians and Europeans may influence the outcome of the association of biomarker with the genetic instrument. However, this would less likely be the case because; 1) the genetic instruments used for T2D and 25(OH)D were from bonafide candidate genes; 2) the application of sample-size weighted Z-score method would reduce inter-study variation, and 3) our metanalysis results do not show much heterogeneity in the SNP-phenotype association across all cohorts.

Additionally, seasonal differences in sun exposure (UV index) for effecting 25(OH)D concentration in most studies were not available except for our Punjabi Sikh cohort (AIDHS/SDS). Nonetheless, the geographic location of most participating European cohorts was nearby (Northern Europe), which might not have confounded the results due to latitude (to derive distance from the equator) variation (which all ranged from 54^0^ to 55^0^ N) (See Supplementary Table 10). Nevertheless, adjusting for the effects of the UV index in our Punjabi sample (AIDHS/SDS) did not change the overall impact of genetically instrumented 25(OH)D on T2D susceptibility in AIDHS/SDS and overall across studies.

It is possible to speculate that the lack of association with 25(OH)D metabolism and transport genes, but the association with synthesis gene (DHCR7) could suggest that any association with T2D may be through a UV-dependent, vitamin D-independent effect as described [39]. The power of capturing the causal association would be improved by the expansion and the refinement of the appropriateness of the genetic instrument to determine causality. Moreover, because of some inherent limitations of Mendelian randomization design, including pleiotropy, buffering effects of environmental and genetic forces (redundancy due to more than one gene), and the effects of developmental compensation, large sample size may be required to logically confirm the causal association of 25(OH)D status with T2D.

## Conclusions

For the first time, we have performed a bidirectional multiethnic Mendelian randomization study to determine the causal relationship between T2D and vitamin D concentrations, including data from the population of South Asians with Europeans. Even though our research has not found strong evidence of causation, it also does not rule out the possible contribution of genetic influence of vitamin D synthesis SNPs in increasing T2D risk. As the long-standing question of the role of vitamin D in influencing the risk of T2D remains unaddressed, our data stresses the need for the population-specific design for future observational and randomized clinical trials on this important and controversial topic of immense clinical importance.

In summary, our bidirectional Mendelian randomization study having data from South Asian Indians with Europeans suggests that genetically instrumented T2D risk may not be causing changes in 25(OH)D levels. However, we cannot entirely exclude the likelihood of DHCR7 genetic variants to influence the risk of T2D.

## Supplementary Information

**Additional file 1: Supplementary Table 1.** Association results of individual T2D SNPs used as genetic instruments in Mendelian randomization for their association with T2D. **Supplementary Table 2.** Association of individual T2D SNPs used as genetic instruments in Mendelian randomization analyses for their association with 25(OH)D concentrations. **Supplementary Table 3.** Association results of individual vitamin D SNPs used as genetic instruments in Mendelian randomization for their association with circulating 25(OH)D concentration. **Supplementary Table 4.** Association results of individual vitamin D SNPs used as genetic instruments in Mendelian randomization for their association with T2D. **Supplementary Table 5.** Association of vitamin D SNPs used as genetic instruments for affecting 25(OH)D concentrations using UV index as a covariate. **Supplementary Table 6.** Association of vitamin D SNPs used as genetic instruments showing their effect on Type 2 diabetes risk using UV index as a covariate. **Supplementary Table 7 A-B.** Results of joint metanalysis of current study and a published study (Lu et al, 2018) showing significant associations of variants in vitamin D candidate genes (GC and DHCR7) for effecting 25(OH)D concentrations. **Supplementary Table 8 A-B.** Results of joint metanalysis of current study and a published study (Lu et al, 2018) showing effects of genetically instrumented vitamin D candidate genes (GC and DHCR7) SNPs for their effects on T2D risk. **Supplementary Table 9.** Information on samples with missing 25(OH)D levels from 8 different cohorts. **Supplementary Table 10.** Differences in latitude, 25(OH)D concentration and distribution of allele frequencies of vitamin D and T2D SNPs among South Asian and European cohorts.

**Additional file 2: Supplementary (Flow Chart).** Experimental plan including the details of participating cohorts and candidate gene SNPs used as genetic instrument for performing a bidirectional Mendelian randomization study. **Supplementary Figure 1 (A-B).** Association analysis of composite gene score of T2D SNPs (IGF2BP2, TCF7L2 and KCNQ1) used as a genetic instrument for their joint effect on T2D and 25(OH)D concentrations (https://www.graphpad.com/scientific-software/prism/). **Supplementary Figure 2 (A-B).** Association analysis of composite gene score of vitamin D SNPs (GC, CYP2R1 and DHCR7) used as a genetic instrument for their effect on 25(OH)D concentrations and T2D (https://www.graphpad.com/scientific-software/prism/). **Supplementary Figure 3.** This figure illustrates pathways of synthesis, absorption, metabolism, and transportation of vitamin D. Genetic factors known to influence circulating levels of vitamin D are shown in the figure. DHCR7 expresses a reductase which uses nicotinamide adenine dinucleotide phosphate-oxidase to catalyzes the production of cholesterol to 7-dehydrocholesterol (7-DHC). The GC gene catalyzes the vitamin D binding protein formation. The enzyme (25-hydroxylase) responsible for the first hydroxylation step is encoded by CYP2R1. The enzyme in the kidneys responsible for the second hydroxylation is catalyzed by the CYP27B1 gene product (not used in this study). 1,25(OH)2D3 is the most effective and commonly measured vitamin D deficiency marker. Genetic instruments used in this study include gene pathways involved in vitamin D synthesis (DHCR7), metabolism (CYP2R1) and transport (GC) and genetic loci linked with increased T2D risk (IGF2BP2, TCF7L2, and KCNQ1). Note that in our results causality appears to be inferred by direct association of 25(OH)D variants (i.e. DHCR7) with 25(OH)D levels and the T2D phenotype. In contrast the T2D genes are associated with phenotype but not with the intermediate phenotype measured as 25(OH)D.

**Additional file 3.** Online Supplemental Information: Phenotypic measurements including T2D, 25(OH)D, and other recruitment details are presented separately for each cohort.

## Data Availability

On request from corresponding author.
