# Supplementary material for: A Bidirectional Mendelian Randomization Study to evaluate the causal role of reduced blood vitamin D levels with type 2 diabetes risk in South Asians and Europeans
Source: Nutr J. 2021 Jul 27;20:71. doi: 10.1186/s12937-021-00725-1 (PMC8314596; doi:10.1186/s12937-021-00725-1)
Supplement: Supplementary file 1 — Additional file 1: Supplementary Table 1. Association results of individual T2D SNPs used as genetic instruments in Mendelian randomization for their association with T2D. Supplementary Table 2. Association of individual T2D SNPs used as genetic instruments in Mendelian randomization analyses for their association with 25(OH)D concentrations. Supplementary Table 3. Association results of individual vitamin D SNPs used as genetic instruments in Mendelian randomization for their association with circulating 25(OH)D concentration. Supplementary Table 4. Association results of individual vitamin D SNPs used as genetic instruments in Mendelian randomization for their association with T2D. Supplementary Table 5. Association of vitamin D SNPs used as genetic instruments for affecting 25(OH)D concentrations using UV index as a covariate. Supplementary Table 6. Association of vitamin D SNPs used as genetic instruments showing their effect on Type 2 diabetes risk using UV index as a covariate. Supplementary Table 7 A-B. Results of joint metanalysis of current study and a published study (Lu et al, 2018) showing significant associations of variants in vitamin D candidate genes (GC and DHCR7) for effecting 25(OH)D concentrations. Supplementary Table 8 A-B. Results of joint metanalysis of current study and a published study (Lu et al, 2018) showing effects of genetically instrumented vitamin D candidate genes (GC and DHCR7) SNPs for their effects on T2D risk. Supplementary Table 9. Information on samples with missing 25(OH)D levels from 8 different cohorts. Supplementary Table 10. Differences in latitude, 25(OH)D concentration and distribution of allele frequencies of vitamin D and T2D SNPs among South Asian and European cohorts. [file 12937_2021_725_MOESM1_ESM.docx]

**Supplementary Table 1. Association results of individual T2D SNPs used as genetic instruments in Mendelian randomization for their association with T2D**

| **T2D_T2D** | | | | | | | | | | | | | | | | | | | |
| --- | --- | --- | --- | --- | --- | --- | --- | --- | --- | --- | --- | --- | --- | --- | --- | --- | --- | --- | --- |
| **IGF2BP2** | **Chr** | **Position** | **SNP** | **Proxy** | **N** | **Other**  **Allele** | **Effect**  **Allele** | **Effect allele frequency** | **OR** | **LCI** | | **UCI** | **P Val** | | | **Z score** | **Direction** | | |
| AIDHS/SDS | 3 | 187011774 | rs1470579 |  | 2709 | C | A | 0.39 | 1.19 | 1.01 | | 1.41 | 1.20E-04 | | |  |  | | |
| SIKH REPLICATION | 3 | 187011774 | rs1470579 |  | 2197 | C | A | 0.38 | 1.13 | 1.01 | | 1.25 | 5.00E-02 | | |  |  | | |
| IMS | 3 | 187011774 | rs1470579 | rs1470580 | 863 | C | A | 0.48 | 1.22 | 0.85 | | 1.77 | 2.75E-01 | | |  |  | | |
| **SA META** | **3** | **187011774** | **rs1470579** |  | **5769** | **C** | **A** | **0.42** | **1.16** | **1.08** | | **1.24** | **8.90E-04** | | | **4.22** | **+++** | | |
| Twins UK | 3 | 187011774 | rs1470579 |  | 5339 | C | A | 0.31 | 1.07 | 0.81 | | 1.43 | 6.26E-01 | | |  |  | | |
| 1958 BC | 3 | 187011774 | rs1470579 |  | 5171 | C | A | 0.31 | 0.95 | 0.68 | | 1.32 | 6.57E-01 | | |  |  | | |
| CCHS/CGPS/CIHDS | 3 | 187011774 | rs1470579 |  | 23036 | C | A | 0.30 | 1.14 | 1.07 | | 1.22 | 1.20E-05 | | |  |  | | |
| UKHLS | 3 | 187011774 | rs1470579 |  | 8905 | C | A | 0.31 | 1.04 | 0.90 | | 1.21 | 5.73E-01 | | |  |  | | |
| PREVEND | 3 | 187011774 | rs1470579 |  | 3649 | C | A | 0.31 | 1.08 | 0.92 | | 1.27 | 1.93E-01 | | |  |  | | |
| **Meta-Analysis** | 3 | 187011774 | **rs1470579** |  | **51869** | **C** | **A** | **0.31** | **1.12** | **1.08** | | **1.16** | **7.21E-07** | | | **4.96** | **++++-+++** | | |
| **Overall phenotypic variance explained 12% (95%CI) 8%-16%** | | | | | | | | | | | | | | | | | | | |
|  |  |  |  |  |  |  |  |  |  | |  |  |  |  |  | |  |  |  |
| **TCF7L2** | **Chr** | **Position** | **SNP** | **Proxy** | **N** | **Other**  **Allele** | **Effect**  **Allele** | **Effect allele frequency** | **OR** | **LCI** | | **UCI** | **P Val** | | | **Z score** | **Direction** | | |
| AIDHS/SDS | 10 | 114748339 | rs7903146 |  | 2709 | T | C | 0.35 | 1.44 | 1.22 | | 1.71 | 6.00E-08 | | |  |  | | |
| SIKH REPLICATION | 10 | 114748339 | rs7903146 |  | 2197 | T | C | 0.33 | 1.48 | 1.40 | | 1.56 | 7.42E-05 | | |  |  | | |
| IMS | 10 | 114748339 | rs7903146 |  | 863 | T | C | 0.31 | 1.00 | 0.66 | | 1.52 | 9.95E-01 | | |  |  | | |
| **SA META** | **10** | **114748339** | **rs7903146** |  | **5769** | **T** | **C** | **0.33** | **1.46** | **1.40** | | **1.52** | **8.64E-12** | | | **6.16** | **+++** | | |
| Twins UK | 10 | 114748339 | rs7903146 |  | 5339 | T | C | 0.28 | 1.30 | 0.95 | | 1.77 | 1.01E-01 | | |  |  | | |
| 1958 BC | 10 | 114748339 | rs7903146 |  | 5171 | T | C | 0.30 | 1.51 | 1.19 | | 1.90 | 5.50E-04 | | |  |  | | |
| CCHS/CGPS/CIHDS | 10 | 114748339 | rs7903146 |  | 23036 | T | C | 0.27 | 1.25 | 1.22 | | 1.28 | 4.50E-13 | | |  |  | | |
| UKHLS | 10 | 114748339 | rs7903146 |  | 8905 | T | C | 0.29 | 1.30 | 1.13 | | 1.50 | 3.50E-04 | | |  |  | | |
| PREVEND | 10 | 114748339 | rs7903146 |  | 3649 | T | C | 0.27 | 1.49 | 1.27 | | 1.76 | 1.00E-06 | | |  |  | | |
| **Meta-Analysis** | 10 | 114748339 | **rs7903146** |  | **51869** | **T** | **C** | **0.28** | **1.25** | **1.23** | | **1.28** | **1.30E-32** | | | **11.31** | **++++++++** | | |
| **Overall phenotypic variance explained 25% (95%CI) 23%-28%** | | | | | | | | | | | | | | | | | | | |
|  |  |  |  |  |  |  |  |  |  | |  |  |  |  |  | |  |  |  |
| **KCNQ1** | **Chr** | **Position** | **SNP** | **Proxy** | **N** | **Other**  **Allele** | **Effect**  **Allele** | **Effect allele frequency** | **OR** | **LCI** | | **UCI** | **P Val** | | | **Z score** | **Direction** | | |
| AIDHS/SDS | 11 | 2815016 | rs2237896 |  | 2709 | A | G | 0.03 | 1.58 | 1.44 | | 1.69 | 2.2E-06 | | |  |  | | |
| SIKH REPLICATION | 11 | 2815016 |  | rs2237892 | 2197 | A | G | 0.02 | 1.31 | 0.82 | | 1.80 | 2.91E-01 | | |  |  | | |
| IMS | 11 | 2815016 | rs2237896 |  | 863 | A | G | 0.01 | 1.91 | 1.08 | | 1.99 | 4.30E-02 | | |  |  | | |
| **SA META** | **11** | **2815016** |  |  | **5769** | **A** | **G** | **0.02** | **1.58** | **1.53** | | **1.64** | **6.41E-06** | | | **4.51** | **+++** | | |
| Twins UK | 11 | 2815016 | rs2237896 | rs2237892 | 5339 | A | G | 0.06 | 1.22 | 0.37 | | 1.63 | 5.03E-01 | | |  |  | | |
| 1958 BC | 11 | 2815016 | rs2237896 |  | 5171 | A | G | 0.04 | 1.24 | 0.22 | | 1.70 | 3.74E-01 | | |  |  | | |
| CCHS/CGPS/CIHDS | 11 | 2815016 | rs2237896 | rs2237897 | 23036 | A | G | 0.04 | 1.17 | 0.89 | | 1.38 | 5.60E-02 | | |  |  | | |
| UKHLS | 11 | 2815016 | rs2237896 | other snp | 8905 | A | G | 0.06 | 1.20 | 0.92 | | 1.41 | 1.53E-01 | | |  |  | | |
| PREVEND | 11 | 2815016 | rs2237896 |  | 3649 | A | G | 0.06 | 1.29 | 0.43 | | 1.68 | 1.35E-01 | | |  |  | | |
| **Meta-Analysis** | 11 | 2815016 | **rs2237896** |  | **51869** | **A** | **G** | **0.05** | **1.49** | **1.43** | | **1.55** | **5.07E-07** | | | **5.02** | **++++++++** | | |
| **Overall phenotypic variance explained 49% (95%CI) 43%-55%** | | | | | | | | | | | | | | | | | | | |

| **T2D_VitD** | | | | | | | | | | | | | | | | |
| --- | --- | --- | --- | --- | --- | --- | --- | --- | --- | --- | --- | --- | --- | --- | --- | --- |
| **IGF2BP2** | **Chr** | **Position** | **SNP** | **Proxy** | **N** | **Other**  **Allele** | **Effect**  **Allele** | **Effect allele frequency** | **Beta** | **SE** | **P Val** | **Z score** | | **Direction** | | |
| AIDHS/SDS | 3 | 185529080 | rs1470579 |  | 2304 | C | A | 0.42 | 0.005 | 0.02 | 8.50E-01 |  | |  | | |
| SIKH REPLICATION | 3 | 185529080 | rs1470579 |  | 2197 | C | A | 0.39 | 0.01 | 0.03 | 6.27E-01 |  | |  | | |
| **SA META** | **3** | **185529080** | **rs1470579** |  | **4501** | **C** | **A** | **0.41** | **0.007** | **0.02** | **6.30E-01** | **0.48** | | **++** | | |
| Twins UK | 3 | 187011774 | rs1470579 |  | 3683 | C | A | 0.31 | 0.010 | 0.01 | 4.70E-01 |  | |  | | |
| 1958 BC | 3 | 187011774 | rs1470579 |  | 4982 | C | A | 0.31 | 0.004 | 0.01 | 6.60E-01 |  | |  | | |
| CCHS/CGPS/CIHDS | 3 | 187011774 | rs1470579 |  | 11667 | C | A | 0.30 | 0.010 | 0.01 | 3.00E-01 |  | |  | | |
| PREVEND | 3 | 185529080 | rs1470579 |  | 3210 | C | A | 0.31 | -0.001 | 0.01 | 9.60E-01 |  | |  | | |
| **Meta-Analysis** | 3 | 185529080 | **rs1470579** |  | **28043** | **C** | **A** | **0.32** | **0.0058** | **0.005** | **2.00E-01** | **1.29** | | **+++++-** | | |
| **Overall phenotypic variance explained 0.58 nmol/L ± 0.5 nmol/L** | | | | | | | | | | | | | | | | |
|  |  |  |  |  |  |  |  |  |  |  |  |  |  | |  |  |
| **TCF7L2** | **Chr** | **Position** | **SNP** | **Proxy** | **N** | **Other**  **Allele** | **Effect**  **Allele** | **Effect allele frequency** | **Beta** | **SE** | **P Val** | **Z score** | | **Direction** | | |
| AIDHS/SDS | 10 | 114758349 | rs7903146 |  | 2304 | T | C | 0.38 | -0.086 | 0.02 | 1.0E-03 |  | |  | | |
| SIKH REPLICATION | 10 | 114758349 | rs7903146 |  | 2197 | T | C | 0.36 | -0.05 | 0.03 | 6.25E-02 |  | |  | | |
| **SA META** | **10** | **114758349** | **rs7903146** |  | **4501** | **T** | **C** | **0.37** | **-0.07** | **0.02** | **2.69E-04** | **-3.64** | | **--** | | |
| Twins UK | 10 | 114748339 | rs7903146 |  | 3683 | T | C | 0.28 | -0.015 | 0.01 | 2.80E-01 |  | |  | | |
| 1958 BC | 10 | 114748339 | rs7903146 |  | 4982 | T | C | 0.30 | 0.001 | 0.01 | 9.30E-01 |  | |  | | |
| CCHS/CGPS/CIHDS | 10 | 114748339 | rs7903146 |  | 11667 | T | C | 0.27 | -0.0004 | 0.01 | 7.70E-01 |  | |  | | |
| PREVEND | 10 | 114758349 | rs7903146 |  | 3210 | T | C | 0.27 | -0.009 | 0.01 | 3.80E-01 |  | |  | | |
| **Meta-Analysis** | 10 | 114758349 | **rs7903146** |  | **28043** | **T** | **C** | **0.29** | **-0.011** | **0.005** | **2.00E-02** | **-2.30** | | **---+--** | | |
| **Overall phenotypic variance explained -1.1 nmol/L ± 0.5 nmol/L** | | | | | | | | | | | | | | | | |
|  |  |  |  |  |  |  |  |  |  |  |  |  |  | |  |  |
| **KCNQ1** | **Chr** | **Position** | **SNP** | **Proxy** | **N** | **Other**  **Allele** | **Effect**  **Allele** | **Effect allele frequency** | **Beta** | **SE** | **P Val** | **Z score** | | **Direction** | | |
| AIDHS/SDS | 11 | 2815016 | rs2237896 |  | 2304 | A | G | 0.02 | -0.027 | 0.07 | 4.80E-01 |  | |  | | |
| SIKH REPLICATION | 11 | 2815016 |  | rs2237892 | 2197 | A | G | 0.02 | -0.07 | 0.06 | 2.80E-01 |  | |  | | |
| **SA META** | **11** | **2815016** |  |  | **4501** | **A** | **G** | **0.02** | **-0.05** | **0.04** | **2.10E-01** | **-1.26** | | **--** | | |
| Twins UK | 11 | 2815016 | rs2237896 | rs2237892 | 3683 | A | G | 0.06 | -0.039 | 0.03 | 1.20E-01 |  | |  | | |
| 1958 BC | 11 | 2815016 | rs2237896 |  | 4982 | A | G | 0.04 | -0.015 | 0.02 | 4.60E-01 |  | |  | | |
| CCHS/CGPS/CIHDS | 11 | 2815016 | rs2237896 | rs2237897 | 11667 | A | G | 0.04 | 0.027 | 0.02 | 1.70E-01 |  | |  | | |
| PREVEND | 11 | 2815016 | rs2237896 |  | 3210 | A | G | 0.06 | -0.002 | 0.03 | 8.50E-01 |  | |  | | |
| **Meta-Analysis** | 11 | 2815016 | **rs2237896** |  | **28043** | **A** | **G** | **0.04** | **-0.005** | **0.01** | **2.60E-01** | **-1.18** | | **----+-** | | |
| **Overall phenotypic variance explained -0.5 nmol/L ± 1 nmol/L** | | | | | | | | | | | | | | | | |

**Supplementary Table 2. Association of individual T2D SNPs used as genetic instruments in Mendelian randomization analyses for their association with 25(OH)D concentrations**

**Supplementary Table 3. Association results of individual vitamin D SNPs used as genetic instruments in Mendelian randomization for their association with circulating 25(OH)D concentration**

| **VitD_VitD** | | | | | | | | | | | | | |
| --- | --- | --- | --- | --- | --- | --- | --- | --- | --- | --- | --- | --- | --- |
| **GC** | **Chr** | **Position** | **SNP** | **Proxy** | **N** | **Other**  **Allele** | **Effect**  **Allele** | **Effect allele frequency** | **Beta** | **SE** | **P Val** | **Z score** | **Direction** |
| AIDHS/SDS | 4 | 72827247 | rs2282679 |  | 2388 | G | T | 0.31 | -0.103 | 0.03 | 1.40E-04 |  |  |
| SIKH REPLICATION | 4 | 72827247 | rs2282679 |  | 1846 | G | T | 0.33 | -0.11 | 0.03 | 3.32E-05 |  |  |
| **SA META** | **4** | **72827247** | **rs2282679** |  | **4234** | **G** | **T** | **0.32** | **-0.11** | **0.02** | **1.83E-08** | **-5.63** | **--** |
| Twins UK | 4 | 72827247 | rs2282679 | rs1155563 | 5339 | G | T | 0.30 | -0.072 | 0.01 | 1.00E-07 |  |  |
| 1958 BC | 4 | 72827247 | rs2282679 |  | 5171 | G | T | 0.29 | -0.082 | 0.01 | 6.90E-20 |  |  |
| CCHS/CGPS/CIHDS | 4 | 72827247 | rs2282679 |  | 11665 | G | T | 0.28 | -0.083 | 0.01 | 1.00E-25 |  |  |
| PREVEND | 4 | 72827247 | rs2282679 |  | 3649 | G | T | 0.27 | -0.125 | 0.01 | 1.00E-07 |  |  |
| **Meta-Analysis** | 4 | 72827247 | **rs2282679** |  | **30058** | G | T | **0.29** | **-0.091** | **0.005** | **2.87E-61** | **-16.52** | **-------** |
| **Overall phenotypic variance explained -9.1 nmol/L ± 0.5 nmol/L** | | | | | | | | | | | | | |

| **CYP2R1** | **Chr** | **Position** | **SNP** | **Proxy** | **N** | **Other**  **Allele** | **Effect**  **Allele** | **Effect allele frequency** | **Beta** | **SE** | **P Val** | **Z score** | **Direction** |
| --- | --- | --- | --- | --- | --- | --- | --- | --- | --- | --- | --- | --- | --- |
| AIDHS/SDS | 11 | 14870151 | rs12794714 |  | 2388 | A | G | 0.39 | -0.064 | 0.02 | 1.50E-02 |  |  |
| SIKH REPLICATION | 11 | 14870151 |  | rs11023332 | 1846 | A | G | 0.43 | -0.02 | 0.03 | 5.52E-01 |  |  |
| **SA META** | **11** | **14870151** |  |  | **4234** | **A** | **G** | **0.41** | **-0.05** | **0.02** | **2.00E-03** | **-2.14** | **--** |
| Twins UK | 11 | 14870151 | rs12794714 | rs11023350 | 5339 | A | G | 0.44 | -0.030 | 0.01 | 2.20E-02 |  |  |
| 1958 BC | 11 | 14870151 | rs12794714 | rs11023350 | 5171 | A | G | 0.44 | -0.025 | 0.01 | 2.30E-03 |  |  |
| CCHS/CGPS/CIHDS | 11 | 14870151 | rs12794714 |  | 31040 | A | G | 0.41 | -0.043 | 0.004 | 1.40E-26 |  |  |
| PREVEND | 11 | 14870151 | rs12794714 |  | 3649 | A | G | 0.42 | -0.034 | 0.01 | 1.40E-03 |  |  |
| **Meta-Analysis** | 11 | 14870151 | **rs12794714** |  | **49433** | A | G | **0.42** | **-0.039** | **0.0032** | **7.56E-34** | **-11.66** | **-------** |
| **Overall phenotypic variance explained -3.9 nmol/L ± 0.32 nmol/L** | | | | | | | | | | | | | |

| **DHCR7** | **Chr** | **Position** | **SNP** | **Proxy** | **N** | **Other**  **Allele** | **Effect**  **Allele** | **Effect allele frequency** | **Beta** | **SE** | **P Val** | **Z score** | **Direction** |
| --- | --- | --- | --- | --- | --- | --- | --- | --- | --- | --- | --- | --- | --- |
| AIDHS/SDS | 11 | 70843273 | rs12785878 |  | 2388 | G | T | 0.73 | -0.046 | 0.03 | 1.03E-01 |  |  |
| SIKH REPLICATION | 11 | 70843273 | rs12785878 |  | 1846 | G | T | 0.73 | -0.06 | 0.03 | 2.00E-02 |  |  |
| **SA META** | **11** | **70843273** | **rs12785878** |  | **4234** | **G** | **T** | **0.73** | **-0.05** | **0.02** | **5.00E-03** | **-2.79** | **--** |
| Twins UK | 11 | 70843273 | rs12785878 | rs3794060 | 5339 | G | T | 0.22 | -0.048 | 0.01 | 1.20E-03 |  |  |
| 1958 BC | 11 | 70843273 | rs12785878 | rs3750997 | 5171 | G | T | 0.22 | -0.034 | 0.01 | 5.30E-04 |  |  |
| CCHS/CGPS/CIHDS | 11 | 70843273 | rs12785878 | rs7944926 | 31040 | G | T | 0.32 | -0.043 | 0.004 | 3.00E-23 |  |  |
| PREVEND | 11 | 70843273 | rs12785878 |  | 3649 | G | T | 0.29 | -0.039 | 0.01 | 1.40E-03 |  |  |
| **Meta-Analysis** | 11 | 70843273 | **rs12785878** |  | **49433** | G | T | **0.32** | **-0.042** | **0.0032** | **9.00E-32** | **-11.73** | **------** |
| **Overall phenotypic variance explained -4.2 nmol/L ± 0.32 nmol/L** | | | | | | | | | | | | | |

**Supplementary Table 4. Association results of individual vitamin D SNPs used as genetic instruments in Mendelian randomization for their association with T2D**

| **VitD_T2D** | | | | | | | | | | | | | | | |
| --- | --- | --- | --- | --- | --- | --- | --- | --- | --- | --- | --- | --- | --- | --- | --- |
| **GC** | **Chr** | **Position** | **SNP** | **Proxy** | **N** | **Other**  **Allele** | **Effect**  **Allele** | **Effect allele frequency** | **OR** | **LCI** | **UCI** | **P Val** | **Z score** | **Direction** |  |
| AIDHS/SDS | 4 | 72827247 | rs2282679 |  | 2388 | G | T | 0.31 | 1.08 | 0.94 | 1.24 | 3.17E-01 |  |  |  |
| SIKH REPLICATION | 4 | 72827247 | rs2282679 |  | 1846 | G | T | 0.34 | 1.12 | 0.99 | 1.23 | 7.00E-02 |  |  |  |
| **SA META** | **4** | **72827247** | **rs2282679** |  | **4234** | **G** | **T** | **0.32** | **1.09** | **1.02** | **1.17** | **2.90E-02** | **1.99** | **++** |  |
| Twins UK | 4 | 72827247 | rs2282679 | rs1155563 | 5339 | G | T | 0.30 | 0.97 | 0.63 | 1.51 | 8.79E-01 |  |  |  |
| 1958 BC | 4 | 72827247 | rs2282679 |  | 5171 | G | T | 0.29 | 0.73 | 0.46 | 1.15 | 1.50E-02 |  |  |  |
| CCHS/CGPS/CIHDS | 4 | 72827247 | rs2282679 |  | 23036 | G | T | 0.28 | 1.01 | 0.92 | 1.11 | 6.54E-01 |  |  |  |
| UKHLS | 4 | 72827247 | rs2282679 |  | 8905 | G | T | 0.29 | 1.02 | 0.84 | 1.25 | 7.65E-01 |  |  |  |
| PREVEND | 4 | 72827247 | rs2282679 |  | 3649 | G | T | 0.27 | 0.96 | 0.76 | 1.22 | 8.46E-01 |  |  |  |
| **Meta-Analysis** | 4 | 72827247 | **rs2282679** |  | **50334** | **G** | **T** | **0.29** | **1.03** | **0.97** | **1.09** | **2.07E-01** | **0.16** | **++--++-** |  |
| **Overall phenotypic variance explained 3% (95%CI) -3%-9%** | | | | | | | | | | | | | | |  |
|  |  |  |  |  |  |  |  |  |  |  |  |  |  |  |  |
| **CYP2R1** | **Chr** | **Position** | **SNP** | **Proxy** | **N** | **Other**  **Allele** | **Effect**  **Allele** | **Effect allele frequency** | **OR** | **LCI** | **UCI** | **P Val** | **Z score** | **Direction** |  |
| AIDHS/SDS | 11 | 14870151 | rs12794714 |  | 2388 | A | G | 0.38 | 1.06 | 0.90 | 1.25 | 6.60E-02 |  |  |  |
| SIKH REPLICATION | 11 | 14870151 |  | rs11023332 | 1846 | A | G | 0.36 | 1.13 | 0.99 | 1.26 | 6.00E-02 |  |  |  |
| **SA META** | **11** | **14870151** |  |  | **4234** | **A** | **G** | **0.37** | **1.09** | **1.00** | **1.19** | **7.30E-02** | **2.63** | **++** |  |
| Twins UK | 11 | 14870151 | rs12794714 | rs11023350 | 5339 | A | G | 0.44 | 0.95 | 0.67 | 1.35 | 7.44E-01 |  |  |  |
| 1958 BC | 11 | 14870151 | rs12794714 | rs11023350 | 5171 | A | G | 0.44 | 1.06 | 0.77 | 1.45 | 6.27E-01 |  |  |  |
| CCHS/CGPS/CIHDS | 11 | 14870151 | rs12794714 |  | 31040 | A | G | 0.41 | 1.00 | 0.95 | 1.05 | 8.97E-01 |  |  |  |
| UKHLS | 11 | 14870151 | rs12794714 |  | 8905 | A | G | 0.43 | 1.00 | 0.84 | 1.20 | 9.71E-01 |  |  |  |
| PREVEND | 11 | 14870151 | rs12794714 |  | 3649 | A | G | 0.42 | 1.03 | 0.83 | 1.27 | 4.32E-01 |  |  |  |
| **Meta-Analysis** | 11 | 14870151 | **rs12794714** |  | **58338** | **A** | **G** | **0.41** | **1.01** | **0.97** | **1.05** | **4.53E-01** | **1.09** | **++-+-++** |  |
| **Overall phenotypic variance explained 1% (95%CI) -3%- 5%** | | | | | | | | | | | | | | |  |
|  |  |  |  |  |  |  |  |  |  |  |  |  |  |  |  |
| **DHCR7** | **Chr** | **Position** | **SNP** | **Proxy** | **N** | **Other**  **Allele** | **Effect**  **Allele** | **Effect allele frequency** | **OR** | **LCI** | **UCI** | **P Val** | **Z score** | **Direction** |  |
| AIDHS/SDS | 11 | 71167449 | rs12785878 |  | 2388 | G | T | 0.73 | 1.02 | 0.85 | 1.22 | 9.01E-01 |  |  |  |
| SIKH REPLICATION | 11 | 71167449 | rs12785878 |  | 1846 | G | T | 0.73 | 1.05 | 0.91 | 1.19 | 4.40E-01 |  |  |  |
| **SA META** | **11** | **71167449** | **rs12785878** |  | **4234** | **G** | **T** | **0.73** | **1.04** | **0.94** | **1.14** | **4.84E-01** | **0.63** | **++** |  |
| Twins UK | 11 | 71167449 | rs12785878 | rs3794060 | 5339 | G | T | 0.23 | 1.31 | 0.75 | 2.28 | 1.24E-01 |  |  |  |
| 1958 BC | 11 | 71167449 | rs12785878 | rs3750997 | 5171 | G | T | 0.22 | 1.19 | 0.79 | 1.78 | 1.88E-01 |  |  |  |
| CCHS/CGPS/CIHDS | 11 | 71167449 | rs12785878 | rs7944926 | 31040 | G | T | 0.32 | 1.05 | 0.97 | 1.13 | 4.30E-02 |  |  |  |
| UKHLS | 11 | 71167449 | rs12785878 |  | 8905 | G | T | 0.22 | 1.04 | 0.83 | 1.30 | 6.48E-01 |  |  |  |
| PREVEND | 11 | 71167449 | rs12785878 |  | 3649 | G | T | 0.29 | 1.04 | 0.88 | 1.24 | 5.12E-01 |  |  |  |
| **Meta-Analysis** | 11 | 71167449 | **rs12785878** |  | **58338** | **G** | **T** | **0.31** | **1.05** | **1.00** | **1.11** | **4.00E-03** | **2.84** | **+++++++** |  |
| **Overall phenotypic variance explained 5% (95%CI) 0% - 11%** | | | | | | | | | | | | | | |  |

**Supplementary Table 5. Association of vitamin D SNPs used as genetic instruments for affecting 25(OH)D concentrations using UV index as a covariate**

| **VitD_VitD** | | | | | | | | | | | | | | | | |
| --- | --- | --- | --- | --- | --- | --- | --- | --- | --- | --- | --- | --- | --- | --- | --- | --- |
| **GC** | **Chr** | **Position** | **SNP** | **Proxy** | **N** | **Alt** | **Ref** | **Freq** | **Beta** | **SE** | **P Val** | **Z score** | **Direction** | | | |
| AIDHS/SDS | 4 | 72827247 | rs2282679 |  | 2675 | G | T | 0.31 | -0.097 | 0.02 | 4E-04 |  |  | | | |
| Twins UK | 4 | 72827247 | rs2282679 | rs1155563 | 5339 | G | T | 0.30 | -0.072 | 0.01 | 1.0E-07 |  |  | | | |
| 1958 BC | 4 | 72827247 | rs2282679 |  | 5171 | G | T | 0.29 | -0.082 | 0.01 | 6.9E-20 |  |  | | | |
| CCHS/CGPS/CIHDS | 4 | 72827247 | rs2282679 |  | 11665 | G | T | 0.28 | -0.083 | 0.01 | 1E-25 |  |  | | | |
| PREVEND | 4 | 72827247 | rs2282679 |  | 3649 | G | T | 0.27 | -0.125 | 0.01 | 1.0E-07 |  |  | | | |
| **Meta-Analysis** | 4 | 72827247 | **rs2282679** |  | **28499** | G | T | **0.28** | **-0.089** | **0.005** | **4.5E-58** | **-16.07** | **-----** | | | |
|  |  |  |  |  |  |  |  |  |  |  |  |  | |  |  |  |
| **CYP2R1** | **Chr** | **Position** | **SNP** | **Proxy** | **N** | **Alt** | **Ref** | **Freq** | **Beta** | **SE** | **P Val** | **Z score** | **Direction** | | | |
| AIDHS/SDS | 11 | 14870151 | rs12794714 |  | 2675 | A | G | 0.38 | -0.076 | 0.02 | 0.003 |  |  | | | |
| Twins UK | 11 | 14870151 | rs12794714 | rs11023350 | 5339 | A | G | 0.44 | -0.030 | 0.01 | 0.022 |  |  | | | |
| 1958 BC | 11 | 14870151 | rs12794714 | rs11023350 | 5171 | A | G | 0.44 | -0.025 | 0.01 | 2.3E-03 |  |  | | | |
| CCHS/CGPS/CIHDS | 11 | 14870151 | rs12794714 |  | 31040 | A | G | 0.41 | -0.043 | 0.004 | 1.4E-26 |  |  | | | |
| PREVEND | 11 | 14870151 | rs12794714 |  | 3649 | A | G | 0.42 | -0.034 | 0.01 | 1.4E-03 |  |  | | | |
| **Meta-Analysis** | 11 | 14870151 | **rs12794714** |  | **47874** | A | G | **0.42** | **-0.039** | **0.003** | **4.7E-33** | **-11.98** | **-----** | | | |
|  |  |  |  |  |  |  |  |  |  |  |  |  | |  |  |  |
| **DHCR7** | **Chr** | **Position** | **SNP** | **Proxy** | **N** | **Alt** | **Ref** | **Freq** | **Beta** | **SE** | **P Val** | **Z score** | **Direction** | | | |
| AIDHS/SDS | 11 | 70843273 | rs12785878 |  | 2675 | G | T | 0.73 | -0.035 | 0.03 | 0.218 |  |  | | | |
| Twins UK | 11 | 70843273 | rs12785878 | rs3794060 | 5339 | G | T | 0.22 | -0.048 | 0.01 | 1.2E-03 |  |  | | | |
| 1958 BC | 11 | 70843273 | rs12785878 | rs3750997 | 5171 | G | T | 0.22 | -0.034 | 0.01 | 5.3E-04 |  |  | | | |
| CCHS/CGPS/CIHDS | 11 | 70843273 | rs12785878 | rs7944926 | 31040 | G | T | 0.32 | -0.043 | 0.004 | 3E-23 |  |  | | | |
| PREVEND | 11 | 70843273 | rs12785878 |  | 3649 | G | T | 0.29 | -0.039 | 0.01 | 1.4E-03 |  |  | | | |
| **Meta-Analysis** |  |  | **rs12785878** |  | **47874** | G | T | **0.30** | **-0.041** | **0.004** | **1.0E-29** | **-11.32** | **-----** | | | |

*Data on UV index were available only in AIDHS/SDS cohort. Additional covariates were age, gender, BMI and T2D where appropriate.

**Supplementary Table 6. Association of vitamin D SNPs used as genetic instruments showing their effect on Type 2 diabetes risk using UV index as a covariate**

| **VitD_T2D** | | | | | | | | | | | | | | | | | | | | | | | | | | | | | |
| --- | --- | --- | --- | --- | --- | --- | --- | --- | --- | --- | --- | --- | --- | --- | --- | --- | --- | --- | --- | --- | --- | --- | --- | --- | --- | --- | --- | --- | --- |
| **GC** | | | **Chr** | **Position** | | **SNP** | | **Proxy** | | | | **N** | | **Alt** | **Ref** | | **Freq** | | **OR** | **LCI** | | **UCI** | | **P Val** | | **Z score** | | **Direction** | |
| AIDHS/SDS | | | 4 | 72827247 | | rs2282679 | |  | | | | 2682 | | G | T | | 0.31 | | 1.06 | 0.93 | | 1.20 | | 0.348 | |  | |  | |
| Twins UK | | | 4 | 72827247 | | rs2282679 | | rs1155563 | | | | 5339 | | G | T | | 0.30 | | 0.97 | 0.63 | | 1.51 | | 0.879 | |  | |  | |
| 1958 BC | | | 4 | 72827247 | | rs2282679 | |  | | | | 5171 | | G | T | | 0.29 | | 0.73 | 0.46 | | 1.15 | | 0.015 | |  | |  | |
| CCHS/CGPS/CIHDS | | | 4 | 72827247 | | rs2282679 | |  | | | | 23036 | | G | T | | 0.28 | | 1.01 | 0.92 | | 1.11 | | 0.654 | |  | |  | |
| UKHLS | | | 4 | 72827247 | | rs2282679 | |  | | | | 8905 | | G | T | | 0.29 | | 1.02 | 0.84 | | 1.25 | | 0.765 | |  | |  | |
| PREVEND | | | 4 | 72827247 | | rs2282679 | |  | | | | 3649 | | G | T | | 0.27 | | 0.96 | 0.76 | | 1.22 | | 0.846 | |  | |  | |
| **Meta-Analysis** | | | 4 | 72827247 | | **rs2282679** | |  | | | | **48775** | | **G** | **T** | | **0.29** | | **1.01** | **0.95** | | **1.08** | | **0.810** | | **-0.24** | | **+--++-** | |
|  |  |  | | |  | |  | |  |  |  | |  | | |  | |  | | |  | |  | |  | |  | |  |
| **CYP2R1** | | | **Chr** | **Position** | | **SNP** | | **Proxy** | | | | **N** | | **Alt** | **Ref** | | **Freq** | | **OR** | **LCI** | | **UCI** | | **P Val** | | **Z score** | | **Direction** | |
| AIDHS/SDS | | | 11 | 14870151 | | rs12794714 | |  | | | | 2675 | | A | G | | 0.38 | | 1.11 | 0.98 | | 1.25 | | 0.078 | |  | |  | |
| Twins UK | | | 11 | 14870151 | | rs12794714 | | rs11023350 | | | | 5339 | | A | G | | 0.44 | | 0.95 | 0.67 | | 1.35 | | 0.744 | |  | |  | |
| 1958 BC | | | 11 | 14870151 | | rs12794714 | | rs11023350 | | | | 5171 | | A | G | | 0.44 | | 1.06 | 0.77 | | 1.45 | | 0.627 | |  | |  | |
| CCHS/CGPS/CIHDS | | | 11 | 14870151 | | rs12794714 | |  | | | | 31040 | | A | G | | 0.41 | | 1.00 | 0.95 | | 1.05 | | 0.897 | |  | |  | |
| UKHLS | | | 11 | 14870151 | | rs12794714 | |  | | | | 8905 | | A | G | | 0.43 | | 1.00 | 0.84 | | 1.20 | | 0.971 | |  | |  | |
| PREVEND | | | 11 | 14870151 | | rs12794714 | |  | | | | 3649 | | A | G | | 0.42 | | 1.03 | 0.83 | | 1.27 | | 0.432 | |  | |  | |
| **Meta-Analysis** | | | 11 | 14870151 | | **rs12794714** | |  | | | | **56779** | | **A** | **G** | | **0.41** | | **1.02** | **0.97** | | **1.07** | | **0.584** | | **0.55** | | **+-++++** | |
|  |  |  | | |  | |  | |  |  |  | |  | | |  | |  | | |  | |  | |  | |  | |  |
| **DHCR7** | | | **Chr** | **Position** | | **SNP** | | **Proxy** | | | | **N** | | **Alt** | **Ref** | | **Freq** | | **OR** | **LCI** | | **UCI** | | **P Val** | | **Z score** | | **Direction** | |
| AIDHS/SDS | | | 11 | 71167449 | | rs12785878 | |  | | | | 2675 | | G | T | | 0.73 | | 0.94 | 0.82 | | 1.09 | | 0.419 | |  | |  | |
| Twins UK | | | 11 | 71167449 | | rs12785878 | | rs3794060 | | | | 5339 | | G | T | | 0.23 | | 1.31 | 0.75 | | 2.28 | | 0.124 | |  | |  | |
| 1958 BC | | | 11 | 71167449 | | rs12785878 | | rs3750997 | | | | 5171 | | G | T | | 0.22 | | 1.19 | 0.79 | | 1.78 | | 0.188 | |  | |  | |
| CCHS/CGPS/CIHDS | | | 11 | 71167449 | | rs12785878 | | rs7944926 | | | | 31040 | | G | T | | 0.32 | | 1.05 | 0.97 | | 1.13 | | 0.043 | |  | |  | |
| UKHLS | | | 11 | 71167449 | | rs12785878 | |  | | | | 8905 | | G | T | | 0.22 | | 1.04 | 0.83 | | 1.30 | | 0.648 | |  | |  | |
| PREVEND | | | 11 | 71167449 | | rs12785878 | |  | | | | 3649 | | G | T | | 0.29 | | 0.96 | 0.76 | | 1.22 | | 0.512 | |  | |  | |
| **Meta-Analysis** | | | 11 | 71167449 | | **rs12785878** | |  | | | | **56779** | | **G** | **T** | | **0.31** | | **1.04** | **0.98** | | **1.11** | | **0.027** | | **2.21** | | **-++++-** | |

*Data on UV index were available only in AIDHS/SDS cohort. Additional covariates were age, gender, BMI and T2D where appropriate.

**Supplementary Table 7 A-B: Results of joint metanalysis of current study and a published study (Lu et al, 2018) showing significant associations of variants in vitamin D candidate genes (GC and DHCR7) for effecting 25(OH)D concentrations**

**A**

| **GC rs2282679** | | | | | | |
| --- | --- | --- | --- | --- | --- | --- |
| **Study** | **N** | **Beta** | **SE** | **P_Val** | **Z score** | **Direction** |
| Current Study | 30058 | -0.091 | 0.005 | 2.87E-61 |  |  |
| Lu et al. (Plos Medicine, 2018) | 359851 | -4.486 | 0.180 | 8.7E-138 |  |  |
| **Meta-Analysis** | **389909** | **-0.094** | **0.005** | **2.73E-189** | **-29.35** | **--** |
| **Phenotyping variance explained -9.4 nmol/L ± 0.5 nmol/L** | | | | | | |

**B**

| **DHCR7 rs12785878** | | | | | | |
| --- | --- | --- | --- | --- | --- | --- |
| **Study** | **N** | **Beta** | **SE** | **P_Val** | **Z score** | **Direction** |
| Current Study | 49433 | -0.042 | 0.003 | 9.00E-32 |  |  |
| Lu et al. (Plos Medicine, 2018) | 428904 | -2.297 | 0.127 | 4.6E-73 |  |  |
| **Meta-Analysis** | **478337** | **-0.043** | **0.003** | **1.26E-98** | **-21.08** | **--** |
| **Phenotyping variance explained -4.3 nmol/L ± 0.3 nmol/L** | | | | | | |

**Supplementary Table 8 A-B: Results of joint metanalysis of current study and a published study (Lu et al, 2018) showing effects of genetically instrumented vitamin D candidate genes (GC and DHCR7) SNPs for their effects on T2D risk.**

**A**

| **GC rs2282679** | | | | | | | |
| --- | --- | --- | --- | --- | --- | --- | --- |
| **Study** | N | **OR** | **LCI** | **UCI** | **P_Val** | **Z score** | **Direction** |
| Current Study | 50334 | 1.03 | 0.97 | 1.09 | 0.207 |  |  |
| Lu et al. (Plos Medicine, 2018) | 359851 | 1.14 | 0.76 | 1.71 | 0.810 |  |  |
| **Meta-Analysis** | **410185** | **1.03** | **0.98** | **1.09** | **0.263** | **1.06** | **++** |
| **Phenotyping variance explained OR 3% (95%CI) -2%-9%** | | | | | | | |

**B**

| **DHCR7 rs12785878** | | | | | | | |
| --- | --- | --- | --- | --- | --- | --- | --- |
| **Study** | **N** | **OR** | **LCI** | **UCI** | **P_Val** | **Z score** | **Direction** |
| Current Study | 58338 | 1.05 | 1.00 | 1.11 | 0.004 |  |  |
| Lu et al. (Plos Medicine, 2018) | 434719 | 1.17 | 1.01 | 1.31 | 0.037 |  |  |
| **Meta-Analysis** | **493057** | **1.07** | **1.02** | **1.13** | **0.0005** | **3.51** | **++** |
| **Phenotyping variance explained 7% (95%CI) 2%-13%** | | | | | | | |

**Supplementary Table 9. Information on samples with missing 25(OH)D levels from 8 different cohorts**

| **Name of the Cohort** | **T2D (available for genotyping)** | **Samples available with Plasma Vit D estimation** | **Missing for Plasma Vit D estimation** |
| --- | --- | --- | --- |
| AIDHS/SDS | 2709 | 2388 | 321 |
| SIKH REPLICATION | 2197 | 1846 | 351 |
| IMS | 863 | NA | 863 |
| TWINS UK | 5339 | 5339 | 0 |
| 1958 BC | 5171 | 5171 | 0 |
| CCHS/CGPS/CIHDS | 23036 | 11665 | 11371 |
| UKHLS | 8905 | NA | 8905 |
| PREVEND | 3649 | 3649 | 0 |
| **TOTAL** | **51869** | **30058** | **21811** |

**Supplementary Table 10. Differences in latitude, 25(OH)D concentration and distribution of allele frequencies of vitamin D and T2D SNPs among South Asian and European cohorts**

| **Area** | **Latitude (⁰N)** | **Mean 25(OH)D nmol/L** | | **GC rs2282679  MAF^2^** | **CYP2R1**  **rs12794714  MAF^2^** | **DHCR7**  **rs12785878 MAF** | **IGF2BP2**  **rs1470579  MAF^2^** | **TCF7L2**  **rs7903146  MAF^2^** | **KCNQ1**  **rs2237896**  **MAF** |
| --- | --- | --- | --- | --- | --- | --- | --- | --- | --- |
| Punjab, India | 31-32 | 37 | 0.31 | | 0.39 | 0.73 | 0.39 | 0.35 | 0.03 |
| London, UK | 55 | 76 | 0.30 | | 0.44 | 0.22 | 0.31 | 0.28 | 0.06 |
| Scotland/ Wales, UK | 55 | 58 | 0.29 | | 0.44 | 0.22 | 0.31 | 0.30 | 0.04 |
| Copenhagen, Denmark | 55 | 54 | 0.28 | | 0.41 | 0.32 | 0.30 | 0.27 | 0.04 |
| Essex, UK | 55 | N/A | 0.29 | | 0.43 | 0.22 | 0.31 | 0.29 | 0.06 |
| Groningen, Netherland | 55 | 59 | 0.27 | | 0.42 | 0.29 | 0.31 | 0.27 | 0.06 |

^1^Latitude based on geographic location of the place of origin of each cohort.

^2^ MAF: minor allele frequency of the 25(OH)D candidate genes.
