# Supplementary material for: A Bidirectional Mendelian Randomization Study to evaluate the causal role of reduced blood vitamin D levels with type 2 diabetes risk in South Asians and Europeans
Source: Nutr J. 2021 Jul 27;20:71. doi: 10.1186/s12937-021-00725-1 (PMC8314596; doi:10.1186/s12937-021-00725-1)
Supplement: Supplementary file 2 — Additional file 2: Supplementary (Flow Chart). Experimental plan including the details of participating cohorts and candidate gene SNPs used as genetic instrument for performing a bidirectional Mendelian randomization study. Supplementary Figure 1 (A-B). Association analysis of composite gene score of T2D SNPs (IGF2BP2, TCF7L2 and KCNQ1) used as a genetic instrument for their joint effect on T2D and 25(OH)D concentrations (https://www.graphpad.com/scientific-software/prism/). Supplementary Figure 2 (A-B). Association analysis of composite gene score of vitamin D SNPs (GC, CYP2R1 and DHCR7) used as a genetic instrument for their effect on 25(OH)D concentrations and T2D (https://www.graphpad.com/scientific-software/prism/). Supplementary Figure 3. This figure illustrates pathways of synthesis, absorption, metabolism, and transportation of vitamin D. Genetic factors known to influence circulating levels of vitamin D are shown in the figure. DHCR7 expresses a reductase which uses nicotinamide adenine dinucleotide phosphate-oxidase to catalyzes the production of cholesterol to 7-dehydrocholesterol (7-DHC). The GC gene catalyzes the vitamin D binding protein formation. The enzyme (25-hydroxylase) responsible for the first hydroxylation step is encoded by CYP2R1. The enzyme in the kidneys responsible for the second hydroxylation is catalyzed by the CYP27B1 gene product (not used in this study). 1,25(OH)2D3 is the most effective and commonly measured vitamin D deficiency marker. Genetic instruments used in this study include gene pathways involved in vitamin D synthesis (DHCR7), metabolism (CYP2R1) and transport (GC) and genetic loci linked with increased T2D risk (IGF2BP2, TCF7L2, and KCNQ1). Note that in our results causality appears to be inferred by direct association of 25(OH)D variants (i.e. DHCR7) with 25(OH)D levels and the T2D phenotype. In contrast the T2D genes are associated with phenotype but not with the intermediate phenotype measured as 25(OH)D. [file 12937_2021_725_MOESM2_ESM.docx]

**Supplementary (Flow Chart)**

**Flow Chart showing Experimental Design**

**59,890 participants from Eight cohorts of European and South Asian Indian ancestry**

**Plasma 25(OH)D measures were available on 30,058 participants**

**SNPs selected from candidate genes identified by GWAS**

**3 T2D SNPs**

**3 Vitamin D SNPs**

***IGF2BP2* (rs1470579)**

***TCF7L2* (rs7903146)**

***KCNQ1* (rs2237896)**

***GC* (rs2282679)**

***CYP2R1* (rs12794714)**

***DHCR7* (rs12785878)**

**Individual SNP Association analysis with 25(OH)D levels and T2D and**

**Genetic instrument Vitamin D**

**25(OH)D Status**

**Genetic instrument T2D**

**T2D Outcome**

**Gene Score Construction**

**Europeans: TWINS UK; 1958 BIRTH COHORT; CCHS/CGPS/CIHDS; UKHLS; PREVEND**

**South Asians: AIDHS/SDS STAGE I; AIDHS/SDS STAGE II; IMS**

***GC* (rs2282679); *CYP2R1* (rs12794714); *DHCR7* (rs12785878)**

***IGF2BP2* (rs1470579); *TCF7L2* (rs7903146); *KCNQ1* (rs2237896)**

**Supplementary Figure 1**

**B**

**A**

**Supplementary Figure 2**

**B**

**A**

**Supplementary Figure 3**

***Transport GC***

Cholesterol

7

-

Dehydrocholesterol

Pre

-

Vitamin D

Vitamin D

***DHCR7***

**UVB**

***CYP2R1***

A

**lifetime**

of direct and pleiotropic effects of genetic

vitamin D deficiency and defective transport

T2D

**T2D GENES**

**25**

**-**

**hydroxy D**

(

intermediate phenotype

)

A

**lifetime**

of effects on T2D

causation

**Vitamin D GENES**


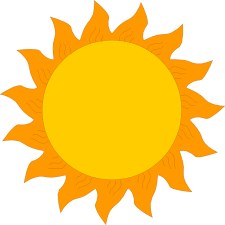


(

***IGFBP2, TCF7L2, KCNQ1***

)

**Hepatic**

**25**

-

**hydroxylation**
