# Supplementary material for: A Bidirectional Mendelian Randomization Study to evaluate the causal role of reduced blood vitamin D levels with type 2 diabetes risk in South Asians and Europeans
Source: Nutr J. 2021 Jul 27;20:71. doi: 10.1186/s12937-021-00725-1 (PMC8314596; doi:10.1186/s12937-021-00725-1)
Supplement: Supplementary file 3 — Additional file 3. Online Supplemental Information: Phenotypic measurements including T2D, 25(OH)D, and other recruitment details are presented separately for each cohort. [file 12937_2021_725_MOESM3_ESM.docx]

**A Bidirectional Mendelian Randomization Study to Evaluate the Causal Role of Reduced Blood Vitamin D Levels With Type 2 Diabetes Risk in South Asians and Europeans**

Cynthia A. Bejar^1^, Shiwali Goyal^1^, Shoaib Afzal^7,8,9^, Massimo Mangino^10,11^, Ang Zhou^12^, Peter J. van der Most ^13^, Yanchun Bao^14^, Vipin Gupta^15^, Melissa C. Smart^14^, Gagandeep K. Walia^16^, Niek Verweij^17^, Christine Power^18^, Dorairaj Prabhakaran^16^, Jai Rup Singh^19^, Narinder K. Mehra^20^, Gurpreet S. Wander^21^, Sarju Ralhan^21^, Sanjay Kinra^22^, Meena Kumari^14^, Martin H. de Borst^17^, Elina Hyppönen^12,18,23^, Tim D. Spector^10^, Børge G. Nordestgaard^7,8,9^, Piers R. Blackett^3,6^, Dharambir K. Sanghera^1,2, ,4,5,6*^

Department of Pediatrics, ^1^Section of Genetics, ^3^Section of Pediatric Endocrinology, College of Medicine, University of Oklahoma Health Sciences Center, Oklahoma City, Oklahoma, USA

^2^Department of Pharmaceutical Sciences, University of Oklahoma Health Sciences Center, Oklahoma City, OK, USA

^4^Department of Physiology, University of Oklahoma Health Sciences Center, Oklahoma City, OK, USA

^5^Oklahoma Center for Neuroscience, University of Oklahoma Health Sciences Center, Oklahoma City, OK, USA

^6^Harold Hamm Diabetes Center, University of Oklahoma Health Sciences Center, Oklahoma City, OK, USA

^7^Department of Clinical Biochemistry, Herlev and Gentofte Hospital, Copenhagen University Hospital, Herlev, Denmark.

^8^The Copenhagen General Population Study, Herlev and Gentofte Hospital, Copenhagen University Hospital, Herlev, Denmark.

^9^Faculty of Health and Medical Sciences, University of Copenhagen, Copenhagen, Denmark

^10^Department of Twin Research and Genetic Epidemiology, Kings College London, London SE1 7EH, UK

^11^NIHR Biomedical Research Centre at Guy’s and St Thomas’ Foundation Trust, London SE1 9RT, UK

^12^Australian Center for Precision Health, University of South Australia Cancer Research Institute, Adelaide, Australia

^13^Department of Epidemiology, University of Groningen, University Medical Center Groningen, Groningen, The Netherlands NL

^14^ Department of Mathematical Sciences, University of Essex, Colchester, UK

^15^Department of Anthropology, University of Delhi, New Delhi, India

^16^Public Health Foundation of India, Gurgaon, India

^17^Department of Internal Medicine, Division of Nephrology, University of Groningen, University Medical Center Groningen, Groningen, The Netherlands

^18^Population, Policy and Practice, Institute of Child Health, University College London, London WC1N 1EH, UK

^19^Department of Human Genetics, Central University of Punjab, Bathinda, Punjab, India

^20^Department of Transplant Immunology and Immunogenetics, All India Institute of Medical Sciences and Research, New Delhi, India

^21^Department of Cardiology, Hero DMC Heart Institute, Ludhiana, India

^22^Department of Non-Communicable Disease Epidemiology, London School of Hygiene and Tropical Medicine, London, UK

^23^Australian Centre for Precision Health, South Australian Health and Medical Research Institute, Adelaide, Australia

**Supplementary Materials**

***Study Cohorts***

**Asian Indian Diabetic Heart Study/Sikh Diabetes Study (AIDHS/SDS)**

The AIDHS/SDS is designed to investigate genetic factors that underlie the unique non-obese metabolically obese phenotype of Punjabi Sikhs. Of these, plasma 25(OH)D concentrations were measured up to 4906 individuals. However, 2709 eligible subjects, those available with genotypes and clinical phenotypes were used in this study for Stage 1. Additional 2197 individuals that were recently genome-wide genotyped were included as replication study. A two stage association analysis of 25(OH)D levels with T2D and genetic variants was carried out in 2709 Punjabi participants in the Stage-1 and 2197 participants in an independent replication cohort (Stage II) from the AIDHS/SDS study, described earlier ([1](#_ENREF_1), [2](#_ENREF_2)). All study subjects in these studies were recruited from the single geographical location of Punjab, India. ([1](#_ENREF_1), [3](#_ENREF_3)). Briefly, the T2D diagnosis was confirmed in all case participants by assessing medical records for symptoms, use of medications, and measuring fasting glucose levels per the American Diabetes Association guidelines ([4](#_ENREF_4)). All subjects with T1D, or who are related to a type 1 diabetic, or have rare forms of T2D subtypes (maturity onset diabetes of the young (MODYs)), or secondary diabetes (from e.g. hemochromatosis, pancreatitis) were excluded from the study. Controls were selected based on fasting blood glucose levels <100.8 mg/dL or a 2-hour glucose <140.0 mg/dL; subjects with impaired fasting glucose (IFG) or impaired glucose tolerance (IGT) were excluded. Body mass index (BMI) was calculated as weight (kg)/ height (m^2^), and obesity was defined using the World Health Organization’s BMI recommendations for Asian populations as described earlier ([5](#_ENREF_5), [6](#_ENREF_6)). Serum was obtained from fasting blood samples to quantify 25(OH)D levels according to PhenX protocol ([7](#_ENREF_7)) (#051100) in the entire AIDHS/SDS cohort (n=3538) using standard monoclonal antibody based ELISA kits from ALPCO Diagnostics (Salem, NH, USA) as described previously ([3](#_ENREF_3), [8](#_ENREF_8), [9](#_ENREF_9)). Samples were blinded for 25(OH)D measurements and each specimen was run in duplicate following the manufacturer’s instructions. A standard curve was used with a range of concentrations (2-fold dilutions) and mixing multiple samples during initial optimization. Any sample that fell out of range was repeated. To minimize batch effect and inter-assay variation across cohorts, samples from both discovery and replication cohorts were quantified using ELISA kits from one manufacturer (ALPCO Diagnostics, Salem, NH, USA) and using one instrument (Tecan Infinite 200 PRO microplate reader). Additional details on plasma vitamin D measurements are described elsewhere([3](#_ENREF_3)). All data were obtained at the baseline visits. All participants provided a written informed consent, and the study was reviewed and approved by the University of Oklahoma Health Sciences Center Institutional Review Board, as well as the Human Subject Protection Committee at the participating hospitals and institutes in India.

**Indian Migration Study (IMS)**

The IMS is a cross sectional study conducted from 2005-2008, aimed at assessing rural-urban differences in prevalence of obesity and diabetes and has been described in detail previously ([10](#_ENREF_10)). Briefly, the urban participants were recruited from factories across four cities in India namely Bangalore, Lucknow, Nagpur, and Hyderabad. Each migrant (urban) factory worker and their spouse were asked to invite one non-migrant full sibling of the same sex and closest to them in age still residing in their rural place of origin to take part in the study. The rural-dwelling siblings came from 18 of the 28 states in India. All the information was collected after receiving informed consent from the study participants. Ethical approval was obtained from the ethics committee of the All India Institute of Medical Sciences, New Delhi, India (IRB# number A-60/4/8/2004). Phenotypic information was available for 7,067 participants, of whom 6,780 individuals were true sib pairs (N=3390 sib-pairs). Out of these, 870 individuals (i.e. 435 sib pairs) were used for the present analyses focused on Type 2 diabetes including 385 discordant and 50 concordant pairs for T2D.

**Twins UK**

The Twins UK registry in St. Thomas’ Hospital, King’s College London recruited a total sample of 11,000 identical and non-identical, mostly female Caucasian, twins from across the UK through national media campaigns. Their age ranges between 16 and 85 years. Over 7,000 twins have attended detailed clinical examinations with a wide range of phenotypes over the last 18 years. All participants were recruited without presence or interest in any particular disease or trait. A total of 2,425 genotyped twins had self-reported information on diabetes. A subset of this dataset (n=5339) had measurement of 25(OH)D and were included in the analysis. This study obtained ethical approval by the Ethics Committee at Guy's and St Thomas Hospital Trust, London. Subjects were not aware of the hypotheses being tested as they were part of a large study investigating many age-related diseases and traits for which informed written consent was obtained (www.twinsuk.ac.uk). 25 (OH)D levels were measured by radioimmunoassay using Diasorin RIA kit (Diasorin, Minnesota, USA) as described previously([11](#_ENREF_11)).

**The British 1958 birth cohort (1958 BC)**

The 1958BC includes all births during one week in March in 1958 in England, Scotland and Wales ([12](#_ENREF_12)). Approximately 17,000 participants were recruited at birth and were subsequently followed up at ages 7, 11, 16, 23, 33, 42, 45, 46 and 50 years. At each follow-up, information on socioeconomic status, health and development, and familial and education factors were obtained. At 45 years of age, 11,971 participants currently living in Britain were invited to take part in a biomedical survey, of whom 9,377 (78%) filled in a questionnaire and 8,302 (89%) also provided a blood sample, in which serum HbA1c and vitamin D concentrations were measured and DNA was extracted for genotyping. Information on type 2 diabetes was obtained by combining self-reported information from surveys at 45, 46 and 50 yrs. Blood 25(OH)D concentrations were measured using automated application of the IDS OCTEIA ELISA on the Dade-Behring BEP2000 analyzer (sensitivity of 5.0nmol/L, linearity ≤155nmol/L, and intra-assay CV 5.5-7.2%), with adjustment according to the mean of the Vitamin D External Quality Assessment Scheme.([13](#_ENREF_13))

**The Copenhagen City Heart Study (CCHS), the Copenhagen General Population Study (CGPS), and the Copenhagen Ischemic Heart Disease Study (CIHDS)**

The CCHS started in 1976-78, with follow-up examinations in 1981-83, 1991-94, and 2001-03. People aged 20-100 were randomly invited from the national Danish Central Person Register to reflect the Danish general population. The CGPS was started in 2003 with ongoing enrolment and with participants recruited as for the CCHS. In CCHS and CGPS cohorts, measurements were carried out using the DiaSorin Liaison 25(OH)D TOTAL assay blinded to outcome and genotypic data ([14](#_ENREF_14), [15](#_ENREF_15)). The CIHDS consists of patients from the Copenhagen area referred for coronary angiography from 1991-2001. We combined data from all three studies as done previously ([14](#_ENREF_14), [16](#_ENREF_16)). Individuals with diagnoses of type 1 or other types of diabetes were excluded. The studies were approved by the Danish ethical committees and Herlev Hospital, Copenhagen University Hospital. Participants gave written informed consent. Analyses included prevalent cases at baseline as well as incident cases.

**The UK Household Longitudinal Study (UKHLS)**

The UKHLS is a panel survey comprising 39802 households across the UK, whose members have been interviewed annually since 2009-10, with eight sweeps completed to date. The study incorporates 8000 households from the British Household Panel Survey, which began in 1991 and comprised 18 data collection sweeps. The study includes phenotypical data for a representative sample of participants for a wide range of social and economic indicators as well as a biological sample collection encompassing biometric, physiological, biochemical, hematological measurements, self-reported medical history and medication use (https://www.understandingsociety.ac.uk/d/100/7251_User_Guide_Health_Assmt_w2_w3.pdf?1392855567). For each participant non-fasting blood samples were collected through venipuncture, were centrifuged to separate plasma and serum, aliquoted and frozen at −80 °C. DNA has been extracted and stored for subsequent genetic analyses, which was performed by the Wellcome Trust Sanger Institute. All participants gave informed consent and ethical approval was granted by the local research ethics committee. Information on how to access the data can be found at https://www.understandingsociety.ac.uk/.

**The Prevention of Renal and Vascular End Stage Disease study (PREVEND)**

The PREVEND study examines the risk factors for and the prevalence and consequences of microalbuminuria in otherwise healthy adults in the city of Groningen([17](#_ENREF_17)). A total of 85,421 people were invited to participate in the study and 40,856 responded. Of these, 8,592 were invited for further screening. Individuals with T1D were excluded. From 1997 on the death certificates of the PREVEND cohort of 8,592 subjects are available. Of these subjects the clinical non-fatal events are also registered using a database of hospital discharge diagnoses. In addition, these subjects are seen every 3-4 years on the outpatient PREVEND facility. Diabetes was defined by any one of the following criteria, if applicable: 1) self-reported diabetes, 2) Fasting blood glucose ≥7.0 mmol/L, 3) HbA1c ≥6.5% (47.5 mmol/ml), or 4) taking antidiabetic medication. BMI was calculated as the ratio between weight (kg) and the square of height (m) (weight/height^2^). Systolic and diastolic BP was calculated as the mean of the last two measurements of the two visits. Circulating 25(OH) and 1,25(OH)2 vitamin D3 levels were measured in baseline plasma samples using liquid chromatography tandem mass spectrometry, with intra and inter-assay coefficients of variation for 25(OH)D of 7.2% and 6.7% ([18](#_ENREF_18))

**SNP Genotyping and Quality Control (QC)**

**AIDHS/SDS**

Genomic DNA was extracted from buffy coats using QiaAmp blood kits (Qiagen, Chatsworth, CA) or by the salting-out procedure as described earlier([19](#_ENREF_19), [20](#_ENREF_20)). We genotyped the discovery set using Human 660W Quad BeadChip panel (Illumina, Inc., San Diego, CA) as explained in Saxena *et al.(*[*2*](#_ENREF_2)*)* We performed pairwise identity-by-state clustering in PLINK across all individuals to assess population stratification; no population outliers were detected. Related individuals with pi-hat 0.3 and samples with, 93% call rate were excluded, as were SNPs with call rate 95%. Also excluded were SNPs with Hardy- Weinberg equilibrium (HWE) P <10^-6^ or minor allele frequency (MAF) <1% as described previously([2](#_ENREF_2)). A total of 1,616 subjects (842 cases and 774 controls) were available for association testing in the discovery cohort for diabetes. Genotyping of replication cohort of 1093 subjects (744 cases and 349 controls) was performed using Illumina’s Global Screening Arrays (GSA) (Illumina, Inc., San Diego, CA). Sample and SNP quality control was performed as described above.

**IMS**

Genomic DNA was isolated from stored whole blood samples using the salt precipitation method at the Centre for Cellular and Molecular Biology, Hyderabad, India. The DNA samples of 5076 IMS participants (2538 sib-pairs) were genotyped using Illumina’s Cardio-Metabochip array at Madras Diabetes Research Foundation using iScan platform. The Metabochip array is a collection of ~200,000 SNPs related to a range of cardiometabolic traits including diabetes and glycemic traits([21](#_ENREF_21)). This array data was utilized to examine association for the genetic variants of T2D in IMS.

**Twins UK**

Genotyping of the TwinsUK dataset was done with a combination of Illumina arrays (HumanHap300 and HumanHap610Q). We pooled the normalized intensity data for each of the arrays separately. For each dataset we used the Illluminus calling algorithm to assign genotypes in the pooled data([22](#_ENREF_22)). No calls were assigned if an individual's most likely genotyped was called with less than a posterior probability threshold of 0.95. Validation of pooling was achieved via a visual inspection of 100 random, shared SNPs for overt batch effects. Finally, intensity cluster plots of significant SNPs were visually inspected for over dispersion biased no calling, and/or erroneous genotype assignment. SNPs exhibiting any of these characteristics were discarded. For the genotype QCs, we applied similar exclusion criteria to each of the dataset separately. Exclusion criteria for the samples were: (i) sample call rate <98%, (ii) heterozygosity across all SNPs ≥2 s.d. from the sample mean; (iii) evidence of non‐European ancestry as assessed by PCA comparison with HapMap3 populations; (iv) observed pairwise IBD probabilities suggestive of sample identity errors. We corrected misclassified monozygotic and dizygotic twins based on IBD probabilities. For the SNPs. We used the following exclusion criteria: (i) Hardy-Weinberg p‐value<10−6, assessed in a set of unrelated samples; (ii) MAF<1%, assessed in a set of unrelated samples; (iii) SNP call rate <97% (SNPs with MAF≥5%) or < 99% (for 1%≤MAF < 5%). Alleles of all datasets were aligned to HapMap2([11](#_ENREF_11)).

**1958 BC**

Genetic information was obtained from blood samples collected at 45 years, through two sub-studies from case-control studies that had used the 1958BC as a source for population controls: 3000 samples were randomly selected as part of the Wellcome Trust Case Control Consortium (WTCCC2 ([23](#_ENREF_23)) and 2592 distinct samples were randomly selected as part of the Type 1 Diabetes Genetics Consortium (T1DGC) ([24](#_ENREF_24)). The WTCCC2 samples were genotyped on the Affymetrix 6.0 platform, whereas T1DGC samples were genotyped using the Illumina Infinium 550 K chip.

**CCHS/CGPS/CIHDS**

Genotypes were derived from the genotyping carried out in the CHD Exome+ Consortium. Briefly, samples were genotyped in batches at the department of clinical biochemistry at Herlev Hospital on a customized version of the Illumina HumanExome v1.1 SNP array. Genotype calling was performed centrally for all batches at the University of Cambridge using optiCall2 (0.7.0), followed by zCall for variants with minor allele frequency (MAF) <5%. The following quality control was performed: intensity outliers, gender mismatch, non-concordance with previous genotyping, duplicate samples or twins, high sample heterozygosity, ancestry outliers in principal components analyses or low call rate as described previously([25](#_ENREF_25)).

**UKHLS**

DNA extracted from samples from 9944 white/European UKHLS participants were genotyped using the HumancoreExome array. Imputation was carried out for SNPs with minor allele frequency of >1% using Minimac to the European component of 1000 Genomes as described([26](#_ENREF_26)).

**Prevend**

The PREVEND cohort was genotyped using Illumina HumanCytoSNP-12 BeadChip (Illumina, San Diego, CA). After quality control as described previously ([27](#_ENREF_27)), genotyped SNPs (MAF >1%) were available for 3,649 subjects (366 cases and 3,283 controls) for association testing. Ungenotyped SNPs were imputed against 1000G phase 1.

**References**

1. Saxena R, Saleheen D, Been LF, Garavito ML, Braun T, Bjonnes A, et al. Genome-Wide Association Study Identifies a Novel Locus Contributing to Type 2 Diabetes Susceptibility in Sikhs of Punjabi Origin From India. Diabetes. 2013;62(5):1746-55.

2. Saxena R, Bjonnes A, Prescott J, Dib P, Natt P, Lane J, et al. Genome-wide association study identifies variants in casein kinase II (CSNK2A2) to be associated with leukocyte telomere length in a Punjabi Sikh diabetic cohort. Circulation Cardiovascular genetics. 2014;7(3):287-95.

3. Sapkota BR, Hopkins R, Bjonnes A, Ralhan S, Wander GS, Mehra NK, et al. Genome-wide association study of 25(OH) Vitamin D concentrations in Punjabi Sikhs: Results of the Asian Indian diabetic heart study. The Journal of steroid biochemistry and molecular biology. 2016;158:149-56.

4. Guidlines ADA. Diagnosis and classification of diabetes mellitus. Diabetes care. 2004;27 Suppl 1:S5-S10.

5. Panel WE. Appropriate body-mass index for Asian populations and its implications for policy and intervention strategies. Lancet. 2004;363(9403):157-63.

6. Been LF, Hatfield JL, Shankar A, Aston CE, Ralhan S, Wander GS, et al. A low frequency variant within the GWAS locus of MTNR1B affects fasting glucose concentrations: Genetic risk is modulated by obesity. Nutr Metab Cardiovasc Dis.

7. McCarty CA, Huggins W, Aiello AE, Bilder RM, Hariri A, Jernigan TL, et al. PhenX RISING: real world implementation and sharing of PhenX measures. BMC medical genomics. 2014;7:16.

8. Sanghera DK, Sapkota BR, Aston CE, Blackett PR. Vitamin D Status, Gender Differences, and Cardiometabolic Health Disparities. Annals of nutrition & metabolism. 2017;70(2):79-87.

9. Braun TR, Been LF, Blackett PR, Sanghera DK. Vitamin D Deficiency and Cardio-Metabolic Risk in a North Indian Community with Highly Prevalent Type 2 Diabetes. Journal of diabetes & metabolism. 2012;3.

10. Ebrahim S, Kinra S, Bowen L, Andersen E, Ben-Shlomo Y, Lyngdoh T, et al. The effect of rural-to-urban migration on obesity and diabetes in India: a cross-sectional study. PLoS medicine. 2010;7(4):e1000268.

11. Wang TJ, Zhang F, Richards JB, Kestenbaum B, van Meurs JB, Berry D, et al. Common genetic determinants of vitamin D insufficiency: a genome-wide association study. Lancet. 2010;376(9736):180-8.

12. Power C, Elliott J. Cohort profile: 1958 British birth cohort (National Child Development Study). International journal of epidemiology. 2006;35(1):34-41.

13. Hypponen E, Turner S, Cumberland P, Power C, Gibb I. Serum 25-hydroxyvitamin D measurement in a large population survey with statistical harmonization of assay variation to an international standard. The Journal of clinical endocrinology and metabolism. 2007;92(12):4615-22.

14. Afzal S, Brondum-Jacobsen P, Bojesen SE, Nordestgaard BG. Genetically low vitamin D concentrations and increased mortality: Mendelian randomisation analysis in three large cohorts. Bmj. 2014;349:g6330.

15. Afzal S, Bojesen SE, Nordestgaard BG. Low 25-hydroxyvitamin D and risk of type 2 diabetes: a prospective cohort study and metaanalysis. Clinical chemistry. 2013;59(2):381-91.

16. Afzal S, Brondum-Jacobsen P, Bojesen SE, Nordestgaard BG. Vitamin D concentration, obesity, and risk of diabetes: a mendelian randomisation study. The lancet Diabetes & endocrinology. 2014;2(4):298-306.

17. Hillege HL, Fidler V, Diercks GF, van Gilst WH, de Zeeuw D, van Veldhuisen DJ, et al. Urinary albumin excretion predicts cardiovascular and noncardiovascular mortality in general population. Circulation. 2002;106(14):1777-82.

18. Keyzer CA, Lambers-Heerspink HJ, Joosten MM, Deetman PE, Gansevoort RT, Navis G, et al. Plasma Vitamin D Level and Change in Albuminuria and eGFR According to Sodium Intake. Clinical journal of the American Society of Nephrology : CJASN. 2015;10(12):2119-27.

19. Sanghera DK, Ortega L, Han S, Singh J, Ralhan SK, Wander GS, et al. Impact of nine common type 2 diabetes risk polymorphisms in Asian Indian Sikhs: PPARG2 (Pro12Ala), IGF2BP2, TCF7L2 and FTO variants confer a significant risk. BMC Med Genet. 2008;9:59.

20. Sanghera DK, Demirci FY, Been L, Ortega L, Ralhan S, Wander GS, et al. PPARG and ADIPOQ gene polymorphisms increase type 2 diabetes mellitus risk in Asian Indian Sikhs: Pro12Ala still remains as the strongest predictor. Metabolism: clinical and experimental. 2010;59(4):492-501.

21. Voight BF, Kang HM, Ding J, Palmer CD, Sidore C, Chines PS, et al. The metabochip, a custom genotyping array for genetic studies of metabolic, cardiovascular, and anthropometric traits. PLoS genetics. 2012;8(8):e1002793.

22. Teo YY, Inouye M, Small KS, Gwilliam R, Deloukas P, Kwiatkowski DP, et al. A genotype calling algorithm for the Illumina BeadArray platform. Bioinformatics. 2007;23(20):2741-6.

23. Wellcome Trust Case Control C. Genome-wide association study of 14,000 cases of seven common diseases and 3,000 shared controls. Nature. 2007;447(7145):661-78.

24. Barrett JC, Clayton DG, Concannon P, Akolkar B, Cooper JD, Erlich HA, et al. Genome-wide association study and meta-analysis find that over 40 loci affect risk of type 1 diabetes. Nature genetics. 2009;41(6):703-7.

25. Myocardial Infarction G, Investigators CAEC, Stitziel NO, Stirrups KE, Masca NG, Erdmann J, et al. Coding Variation in ANGPTL4, LPL, and SVEP1 and the Risk of Coronary Disease. The New England journal of medicine. 2016;374(12):1134-44.

26. Prins BP, Kuchenbaecker KB, Bao Y, Smart M, Zabaneh D, Fatemifar G, et al. Genome-wide analysis of health-related biomarkers in the UK Household Longitudinal Study reveals novel associations. Scientific reports. 2017;7(1):11008.

27. Jansen H, Stolk RP, Nolte IM, Kema IP, Wolffenbuttel BH, Snieder H. Determinants of HbA1c in nondiabetic Dutch adults: genetic loci and clinical and lifestyle parameters, and their interactions in the Lifelines Cohort Study. Journal of internal medicine. 2013;273(3):283-93.
